# A quantitative map of human Condensins provides new insights into mitotic chromosome architecture

**DOI:** 10.1083/jcb.201801048

**Published:** 2018-07-02

**Authors:** Nike Walther, M. Julius Hossain, Antonio Z. Politi, Birgit Koch, Moritz Kueblbeck, Øyvind Ødegård-Fougner, Marko Lampe, Jan Ellenberg

**Affiliations:** 1 Cell Biology and Biophysics Unit, European Molecular Biology Laboratory, Heidelberg, Germany; 2 Advanced Light Microscopy Facility, European Molecular Biology Laboratory, Heidelberg, Germany

## Abstract

The two Condensin complexes in human cells are essential for mitotic chromosome structure. We used homozygous genome editing to fluorescently tag Condensin I and II subunits and mapped their absolute abundance, spacing, and dynamic localization during mitosis by fluorescence correlation spectroscopy (FSC)–calibrated live-cell imaging and superresolution microscopy. Although ∼35,000 Condensin II complexes are stably bound to chromosomes throughout mitosis, ∼195,000 Condensin I complexes dynamically bind in two steps: prometaphase and early anaphase. The two Condensins rarely colocalize at the chromatid axis, where Condensin II is centrally confined, but Condensin I reaches ∼50% of the chromatid diameter from its center. Based on our comprehensive quantitative data, we propose a three-step hierarchical loop model of mitotic chromosome compaction: Condensin II initially fixes loops of a maximum size of ∼450 kb at the chromatid axis, whose size is then reduced by Condensin I binding to ∼90 kb in prometaphase and ∼70 kb in anaphase, achieving maximum chromosome compaction upon sister chromatid segregation.

## Abstract (precis)

Walther et al. systematically fluorescently tag endogenous Condensin subunits and map their abundance, physical spacing, and mitotic dynamics by fluorescence correlation spectroscopy–calibrated live-cell imaging and superresolution microscopy. They propose a three-step hierarchical loop model of mitotic chromosome compaction.

## Introduction

A fundamental structural and functional change of the human genome is the compaction of replicated interphase chromatin into rod-shaped mitotic chromosomes. This process of mitotic chromosome condensation is essential for faithful genome partitioning (Hudson et al., 2009) and involves two conserved structural maintenance of chromosomes (SMC) protein complexes, Condensins I and II (Hirano and Mitchison, 1994; Strunnikov et al., 1995; Hirano et al., 1997; Ono et al., 2003; Yeong et al., 2003). Condensins consist of two shared subunits (SMC2 and SMC4) and three isoform-specific subunits: a kleisin (CAP-H or CAP-H2) and two HEAT-repeat proteins (CAP-D2 or CAP-D3 and CAP-G or CAP-G2). SMC2 and SMC4 are backfolded into long coiled-coils, bringing their N and C termini together into two ATPase domains, and are connected at their central domains, creating a “hinge” between the two subunits. The ATPase domains are bridged by the kleisin and associated HEAT-repeat subunits to form a pentameric ring-like architecture with an estimated length of overall ∼60 nm for the human complexes (Anderson et al., 2002). The kleisin and HEAT-repeat subunits have recently been shown to bind DNA in a unique safety belt arrangement (Kschonsak et al., 2017), and the complexes can progressively move on DNA as motors in vitro (Terakawa et al., 2017), which is consistent with the hypothesis that they actively form and stabilize DNA loops (Nasmyth, 2001; Alipour and Marko, 2012; Goloborodko et al., 2016a,b).

Within the cell, Condensin II is located in the nucleus and has access to chromosomes throughout the cell cycle, whereas Condensin I is cytoplasmic during interphase and can only localize to mitotic chromosomes after nuclear envelope breakdown (NEBD) in prometaphase (Ono et al., 2003, 2004; Hirota et al., 2004; Gerlich et al., 2006). Consistent with this distinct subcellular localization, RNA interference and protein depletion experiments have proposed that the two Condensin isoforms promote different aspects of mitotic chromosome compaction, with Condensin II promoting axial shortening in prophase and Condensin I compacting chromosomes laterally in prometaphase and metaphase (Ono et al., 2003, 2004; Hirota et al., 2004; Green et al., 2012). Both Condensins localize to the longitudinal axis of mitotic chromosomes and are part of the insoluble nonhistone scaffold (Maeshima and Laemmli, 2003; Ono et al., 2003).

Extensive structural, biochemical, cell biological, and molecular biological research over the last two decades led to numerous models about how Condensins may shape mitotic chromosomes (Cuylen and Haering, 2011; Hirano, 2012, 2016; Kschonsak and Haering, 2015; Piskadlo and Oliveira, 2016; Uhlmann, 2016; Kalitsis et al., 2017; Kinoshita and Hirano, 2017). Condensins have been proposed to make topological linkages between two regions within the same chromatid (Cuylen et al., 2011) and thereby introduce loops in the DNA molecule, which, according to the loop-extrusion theory (Nasmyth, 2001; Alipour and Marko, 2012; Goloborodko et al., 2016a,b) and very recent evidence in vitro (Ganji et al., 2018), compact mitotic chromosomes and contribute to their mechanical stabilization (Gerlich et al., 2006; Houlard et al., 2015). However, how such Condensin-mediated linkages could organize the hundreds of megabase-sized DNA molecules of a human chromosome, and how Condensins I and II mediate different aspects of the overall compaction process is still poorly understood. A key requirement to formulate realistic mechanistic models is to know the copy number and stoichiometry as well as the precise spatial arrangement of Condensins I and II within a mitotic chromatid. However, such quantitative data about Condensins in single dividing cells are currently missing.

To address this gap in our knowledge, we set out to quantitatively determine the dynamic association of Condensins I and II with chromosomes throughout mitosis and resolve their spatial organization relative to the axis of single chromatids. To this end, we took advantage of genome editing in human cells to create homozygous fluorescent knock-ins for SMC, kleisin, and HEAT-repeat subunits of both Condensins. We then used fluorescence correlation spectroscopy (FCS)-calibrated live-cell imaging to determine the number of Condensin subunits on chromosomes over the course of mitosis. Furthermore, we used stimulated emission depletion (STED) superresolution microscopy of single chromatids in specific mitotic stages to investigate the axial organization of Condensin complexes. By measuring the physical length of mitotic chromosomes and normalizing it to the genomic length, our comprehensive quantitative imaging–based data of human Condensin complexes on mitotic chromosomes allow us for the first time to formulate models of mitotic chromosome structure that are based on concentrations, physical distances, and mean genomic spacing of the key protein complexes that structure DNA during mitosis. Based on these data, we propose a three-step hierarchical loop-formation model for mitotic chromosome compaction that makes quantitative predictions about the hierarchy and size of loops formed in the chromosomal DNA to achieve accurate genome partitioning during cell division.

## Results and discussion

### Quantitative imaging of five Condensin subunits

To quantitatively investigate the dynamic subcellular distribution of Condensin complexes during mitosis, we generated genome-edited human HeLa Kyoto (HK) cell lines in which all alleles of the endogenous genes for the shared subunit SMC4 and two pairs of kleisin and HEAT-repeat subunits specific to Condensin I (CAP-H and CAP-D2) or Condensin II (CAP-H2 and CAP-D3) were tagged with mEGFP (Fig. S1, A and B). Specific and homozygous tagging was verified with a multistep quality control pipeline (Mahen et al., 2014; Koch et al., 2018; for details, see Materials and methods), ensuring that the tagged subunit was expressed at physiological levels and incorporated into complexes during interphase and mitosis (Fig. S1 B), as well as that mitotic progression and timing were not perturbed (Fig. S1 C), thus indicating full functionality of the fusion proteins.

**Figure 1.**
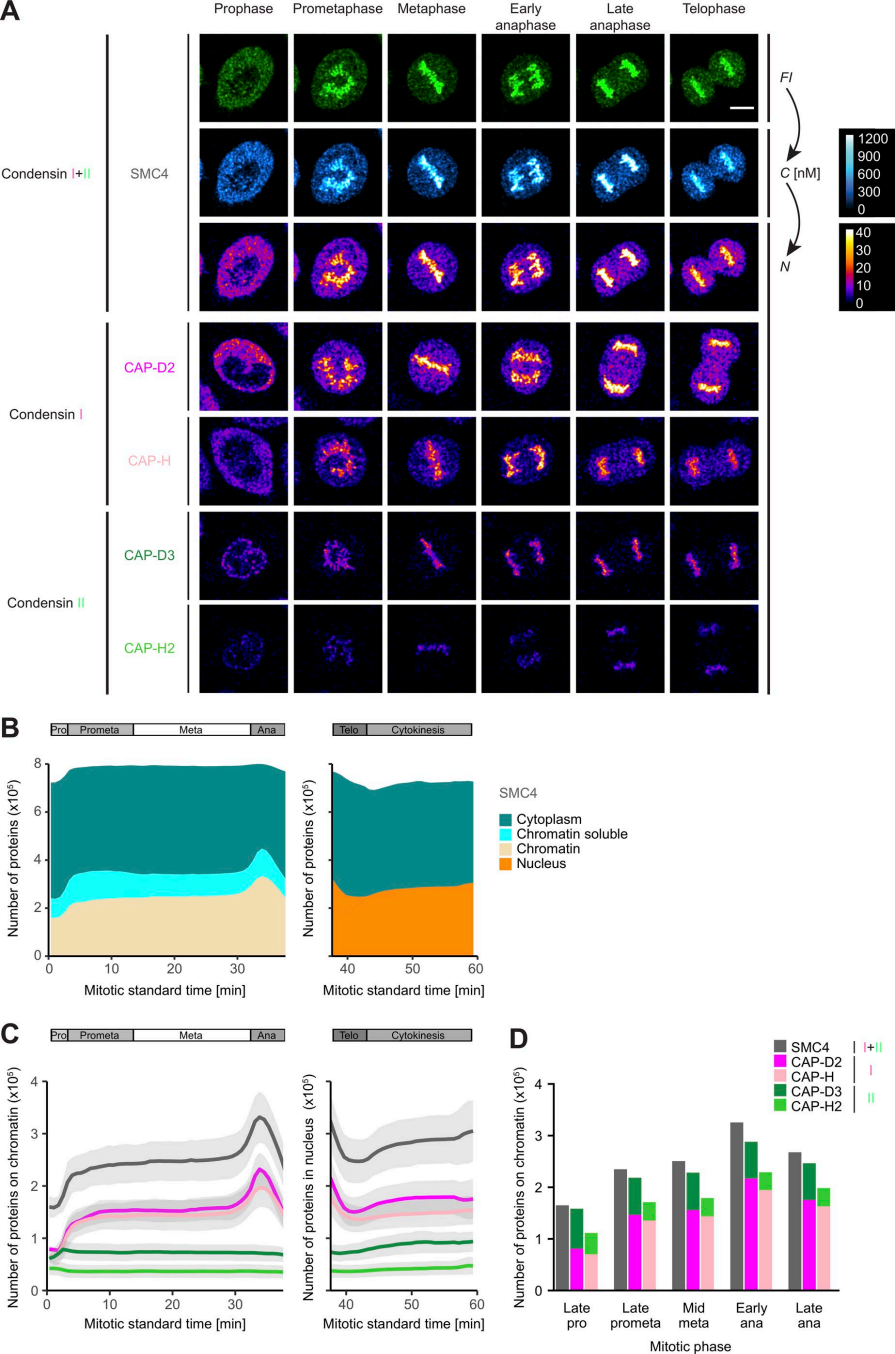
**Condensins I and II display different quantitative dynamics during mitosis as determined by automated FCS-calibrated confocal 3D time-lapse imaging. (A)** Genome-edited HK cells with homozygously mEGFP-tagged Condensin subunits and chromosomes stained by SiR-DNA were imaged every 90 s for a total of 60 min by 3D confocal microscopy, which was automatically triggered after prophase onset. Images were calibrated by FCS to convert fluorescence intensities (*FI*) into cellular protein concentration maps (*C*) and cellular protein number maps (*N*; for details, see Fig. S1, D–I). For SMC4 (Condensin I/II), fluorescence intensities, cellular protein concentration, and protein number maps are shown. For CAP-D2 and CAP-H (Condensin I) and CAP-D3 and CAP-H2 (Condensin II), protein number maps are depicted. Single z planes are shown for specific mitotic phases comparable between subunits. Condensin II is nuclear/on chromosomes throughout mitosis. Condensin I is cytoplasmic in prophase and gains access to mitotic chromosomes after NEBD in prometaphase. A Gaussian blur (σ = 1) was applied to the images for presentation purposes. Bar, 10 µm. **(B)** SMC4 protein numbers in specific compartments of mitotic HK cells (cytoplasm [blue-green], nucleus [orange], chromatin bound [beige], and chromatin soluble [turquoise]) are plotted against the mitotic standard time, and the corresponding mitotic phases are indicated. Shown is the mean of 17 cells from four independent experiments. Cellular landmarks were used for 3D segmentation and conversion of compartment-specific protein concentrations into protein numbers (for details, see Fig. S1 I and Materials and methods). Chromosome-bound and soluble chromatin proteins defined as the freely diffusing proteins within the chromatin volume were distinguished until anaphase as described in Materials and methods. For late mitotic stages, the nuclear compartment could not be divided into chromatin-bound and soluble proteins. **(C)** The numbers of chromosome-bound (prophase to anaphase) and nuclear Condensin subunits (telophase and cytokinesis) are plotted against the mitotic standard time: Condensin II subunits (CAP-D3, dark green; CAP-H2, light green), Condensin I subunits (CAP-D2, dark magenta; CAP-H, light magenta), and shared SMC4 (dark gray) are shown. Means (colored lines) and SD (light gray areas) are shown. **(D)** The numbers of chromosome-bound Condensin subunits from C are plotted for selected mitotic stages according to the images shown in A. SMC4 subunits (gray) as well as the sum of corresponding HEAT-repeat subunits (CAP-D2, dark magenta, Condensin I; CAP-D3, dark green, Condensin II) and corresponding kleisin subunits (CAP-H, light magenta, Condensin I; CAP-H2, light green, Condensin II) are shown. For C and D, the means of ∼20 cells per subunit (range: 10–36) from three to seven independent experiments are plotted; 36, 22, 10, 17, and 17 cells from seven, three, five, three, and four experiments for CAP-H, CAP-H2, CAP-D2, CAP-D3, and SMC4, respectively, are shown.

We then applied automated FCS-calibrated confocal time-lapse microscopy to image Condensin subunits during mitosis relative to spatiotemporal landmarks that defined cell (extracellular Dextran-Dy481XL) and chromatin volumes (DNA stained by SiR-DNA; Lukinavičius et al., 2015; Cai et al., 2017). This calibrated 4D imaging data allowed us to derive time-resolved 3D maps of subcellular protein concentration and copy number of Condensin subunits (Figs. 1 A and S1, D–I; Politi et al., 2018) and distinguish the soluble cytoplasmic from the chromatin-bound protein fractions for all mitotic stages until nuclear envelope reformation (Fig. 1 B; for details, see Materials and methods; Cai et al., 2017). Using computational temporal alignment (Cai et al., 2017), we integrated the data for all Condensin subunits, allowing us to perform a quantitative comparison of their association with mitotic chromosomes (Fig. 1 C).

### About 200,000 Condensin I complexes bind in two steps to mitotic chromosomes

We first examined the total number of all Condensins (SMC4) as well as the number of Condensin I (CAP-D2/CAP-H) and Condensin II (CAP-D3/CAP-H2) subunits in the whole cell (Fig. S2 A), in the cytoplasm (Fig. S2 B), and on chromosomes (Fig. 1 C) throughout mitosis. In the whole cell, the number of Condensin I (∼675,000 for CAP-D2) and Condensin II (∼115,000 for CAP-D3) and correspondingly also Condensin I plus II molecules (∼780,000 for SMC4; Figs. 1 B and S2 A) remained constant throughout mitosis, which indicates that neither new protein synthesis nor protein degradation are used to regulate their function during cell division.

However, the number of Condensin I subunits associated with mitotic chromosomes changed dramatically (Fig. 1 C). With the major fraction gaining access to chromosomes only upon NEBD, the level of chromosome-bound Condensin I reached a first plateau during prometaphase and metaphase, with ∼150,000 CAP-D2 molecules per replicated genome. Upon anaphase onset, a second wave of Condensin I subunits bound to mitotic chromosomes, which resulted in a maximum value of ∼215,000 CAP-D2 molecules on all chromosomes. After chromosome segregation had been accomplished, the number of Condensin I subunits declined rapidly to ∼125,000 CAP-D2 molecules at telophase. Condensin II subunits, in contrast, were much less abundant on chromosomes, with only ∼70,000 CAP-D3 molecules per replicated genome, a number that stayed constant throughout mitosis (Fig. 1 C).

Although we recorded each subunit in individual homozygous knock-in cell lines, the numbers of chromatin-localized isoform-specific subunits (e.g., ∼150,000 CAP-D2 [Condensin I] and ∼70,000 CAP-D3 [Condensin II] in metaphase) summed up very close to the number of chromosome-bound copies of the shared SMC4 subunit (∼250,000 in metaphase) throughout mitosis (Fig. 1 D), validating our cell line generation and quantitative imaging pipelines. The number of ∼250,000 SMC4 subunits on metaphase chromosomes corresponded to a concentration of ∼520 nM on chromatin (Fig. S2 C), whereas the cytoplasmic concentration of ∼190 nM from the ∼530,000 SMC4 molecules in this larger compartment was lower than the chromosomal concentration (Fig. S2 D).

As Condensin I is more abundant than Condensin II, the two-step binding observed for its kleisin and HEAT-repeat subunits was also reflected in the dynamics of the shared SMC4 subunit (Fig. 1, B and C). This behavior is in line with previous studies based on immunostaining or overexpression (Ono et al., 2003, 2004; Hirota et al., 2004; Gerlich et al., 2006).

Our quantitative dynamic imaging data suggest that Condensin II is bound to mitotic chromosomes in prophase and might promote the initial mitotic chromosome compaction, whereas Condensin I is mostly cytoplasmic (Fig. 1 A; Gerlich et al., 2006). The majority of Condensin I rapidly localizes to chromosomes in prometaphase and in a second step in anaphase (Gerlich et al., 2006; Mora-Bermúdez et al., 2007), when it likely promotes further compaction of mitotic chromatids. In the future, it will be very interesting to study how the two waves of Condensin I binding to mitotic chromosomes from a constant cellular pool are regulated during mitosis.

### The kleisin subunit appears to be limiting for Condensin holocomplexes on chromosomes

Interestingly, although they showed very similar dynamics, the number of kleisin subunits for Condensin I and II (CAP-H and CAP-H2) cell lines was consistently lower than the number of the corresponding HEAT-repeat subunits (CAP-D2 and CAP-D3; Fig. 1, C and D), indicating that some chromosome-localizing SMC and HEAT-repeat subunits are missing kleisin subunits to close the pentameric ring, as also suggested in earlier biochemical studies (Kimura et al., 2001; Ono et al., 2003). We confirmed this with both C- and N-terminally tagged CAP-H/H2 subunits and several homozygous clones (Fig. S2, F and G; Cai et al., 2017). For Condensin II, about half of the chromosome-bound CAP-D3 subunits could form holocomplexes (Fig. S2 G), whereas for Condensin I, ∼90% of the CAP-D2 subunits would find sufficient kleisin binding partners for holocomplexes (Fig. S2 F). Based on the limiting kleisin subunits, we estimated a maximal number of ∼35,000 Condensin II and ∼195,000 Condensin I holocomplexes on anaphase chromosomes (Fig. 1 C) and used the kleisin subunits as indicators for Condensin holocomplexes hereafter. Our finding that there were about twice as many chromosome-localizing CAP-D3 subunits than CAP-H2 subunits suggests that either the stoichiometry of CAP-D3 to CAP-H2 in Condensin II complexes is >1:1 or that CAP-D3 can bind mitotic chromosomes independently of being part of Condensin II complexes. Consistent with the latter possibility, precipitating CAP-H2–mEGFP from our endogenously tagged cell lines revealed an unbound fraction of CAP-D3 in the supernatant (SN).

### Condensin I is up to six times more abundant on mitotic chromosomes than Condensin II

Comparison of the number of kleisin subunits as indicators for the maximum amount of holocomplexes at the same mitotic stage revealed that the ratio of Condensin I to Condensin II was ∼4:1 on metaphase chromosomes and increases to ∼5.6:1 in early anaphase (Fig. 1 C), which is consistent with an earlier biochemical assessment in *Xenopus laevis* egg extracts (Ono et al., 2003). The much higher abundance and mitosis-specific binding during the two major stages of compaction suggest that Condensin I is the major contributor to the formation of mitotic chromosomes, consistent with it being required and, together with topoisomerase II, sufficient for mitotic chromosome assembly in vitro (Shintomi et al., 2015, 2017).

Overall, our quantitative FCS-calibrated live-cell imaging databased on homozygous endogenously tagged Condensin subunits revealed that Condensin I is 1.6–5.6× more abundant than Condensin II during mitosis and binds to mitotic chromosomes in two steps in prometaphase and early anaphase. In contrast, the less-abundant Condensin II does not change its association with chromosomes during mitosis, suggesting very different roles for the two Condensin complexes in the structural organization of mitotic chromosomes.

### Condensin I interacts dynamically with mitotic chromosomes, whereas Condensin II binds stably

To investigate how stable the two Condensins are bound to mitotic chromosomes, we investigated their residence times by FRAP (Fig. S2, H–J; for details, see Materials and methods). This revealed that the stepwise association of Condensin I in prometaphase and anaphase and its subsequent rapid decrease in telophase was underpinned by a relatively short chromosomal residence time of ∼2 min (Fig. S2 K) and a low immobile fraction (<20%; Fig. S2 L) on metaphase chromosomes. In contrast, Condensin II was more stably bound with a longer residence time (>5 min; Fig. S2 K) and a much higher immobile fraction (>60%; Fig. S2 L), consistent with its constant abundance on mitotic chromosomes (Fig. 1, C and D). Within 10 min of metaphase, ∼85% of chromosome-bound Condensin I and ∼30% of chromosome-bound Condensin II were exchanged. These findings with endogenously tagged Condensins confirm previous research based on overexpressed proteins (Gerlich et al., 2006). The high turnover of Condensin I binding on mitotic chromosomes is in line with a relatively high cytoplasmic fraction (>60% of the total cellular protein; Fig. S2 E) in contrast to Condensin II, for which the large majority is bound to chromosomes (Fig. S2 E) and exchanges much more slowly. Interestingly, this behavior is consistent between CAP-H2 and CAP-D3. Because the abundance measurements and immunoprecipitations (IPs) indicated that some CAP-D3 proteins can bind chromosomes without being associated with Condensin II complexes (Fig. 1, C and D; and Fig. S2 F), this fraction would then bind with a residence time that cannot be distinguished from CAP-H2 by FRAP. The slow binding dynamics and stable abundance of Condensin II on mitotic chromosomes (Fig. 1, C and D) suggest a more structural and stabilizing role, whereas the dynamic stepwise binding and dissociation of Condensin I is consistent with it playing an actively regulated role in both mitotic compaction and decompaction of chromosomes.

### Superresolution imaging of Condensins I and II in prometaphase and anaphase

Because Condensins I and II display very different chromosome-binding dynamics and stoichiometries in mitosis, we investigated whether they also differ in their subchromosomal localization. To this end, we used superresolution (STED) microscopy to acquire 3D images and localize our tagged Condensin subunits relative to DNA within single chromatids in cells fixed in specific mitotic stages (Fig. S3 A; for details, see Materials and methods). We focused on late prometaphase because at this stage, Condensin I has reached its first binding plateau (Fig. 1 C), and sister chromatids are already resolved (Nagasaka et al., 2016), as well as on early anaphase, when Condensin I abundance peaks (Fig. 1 C) and chromatids reach their maximum compaction (Mora-Bermúdez et al., 2007). Using exactly the same labeling and imaging conditions to visualize the different Condensin subunits allowed us to quantitatively compare and integrate the data between the two isoforms.

**Figure 2.**
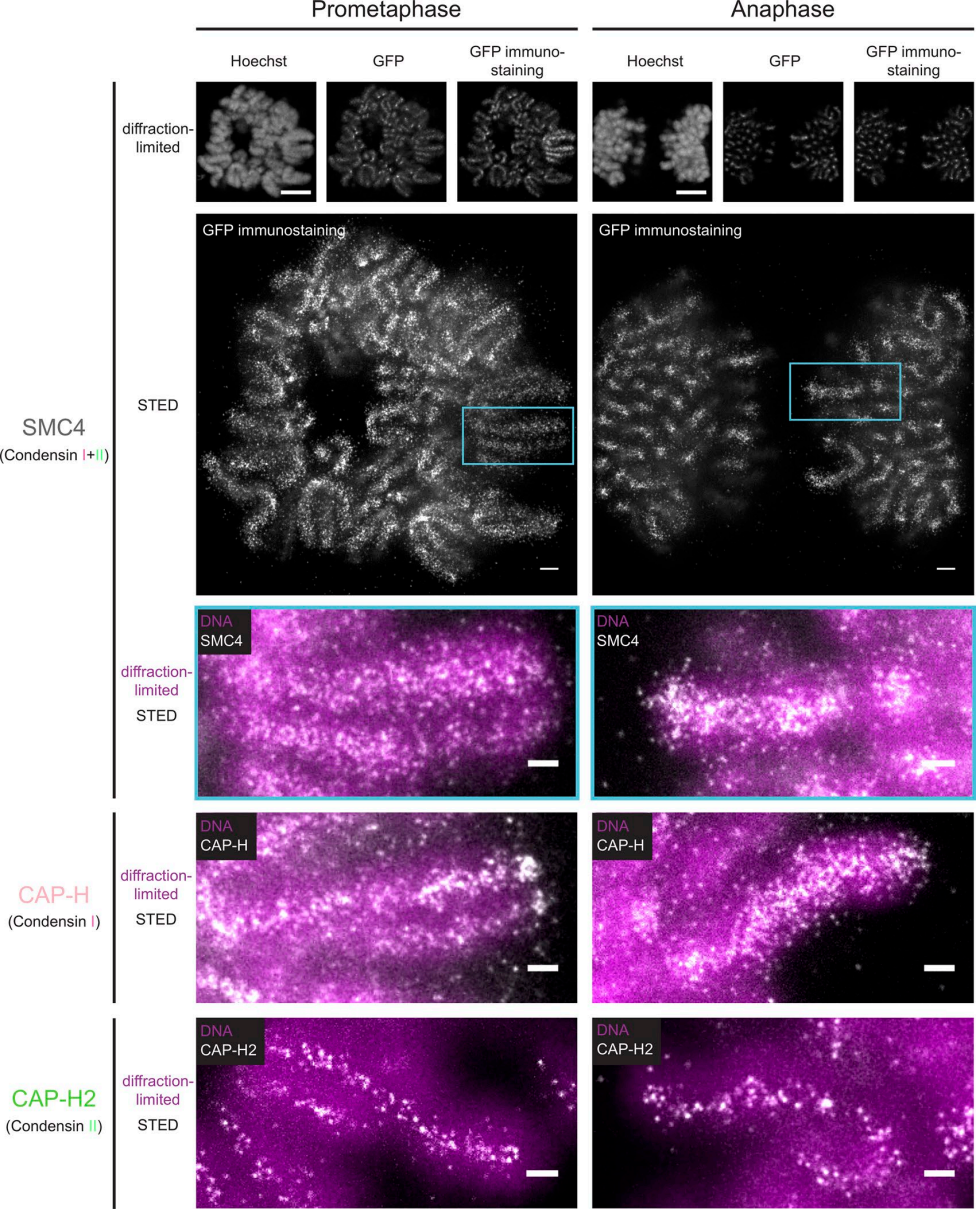
**STED superresolution imaging of Condensins I and II.** Condensin subunits were immunostained (anti-GFP), and mitotic cells with chromatids oriented in parallel to the focal plane were selected for imaging. DNA (Hoechst) and Condensins (mEGFP tag and anti-GFP immunostaining) were imaged by diffraction-limited microscopy, whereas immunostained Condensin was also imaged by STED microscopy. Representative images of mitotic chromatids with SMC4 (Condensin I + II), CAP-H (Condensin I), and CAP-H2 (Condensin II) in late prometaphase (left) and early anaphase (right) are shown. For SMC4 (top), whole-cell overview images as well as overlays of superresolved Condensin (white) and diffraction-limited DNA (magenta) imaging are shown. Overlays represent zooms into single chromatids (turquoise box). For CAP-H (middle) and CAP-H2 (bottom), only overlays are depicted. Representative images of single z planes are shown. Bars: (whole-cell diffraction-limited microscopy) 5 µm; (whole-cell STED microscopy) 1 µm; (zooms) 500 nm.

Our STED data revealed that both Condensins localized along the longitudinal axis in the center of chromatid arms in both prometaphase and anaphase (Fig. 2), consistent with earlier studies (Maeshima and Laemmli, 2003; Ono et al., 2003; Poonperm et al., 2015). In prometaphase, Condensin I decorated chromatids more densely than Condensin II and became even more densely localized in anaphase, consistent with Condensin I’s higher abundance and increased binding as observed by FCS-calibrated imaging in live cells (Fig. 1, C and D).

### Condensin II is more confined to the chromatid axis than Condensin I

**Figure 3.**
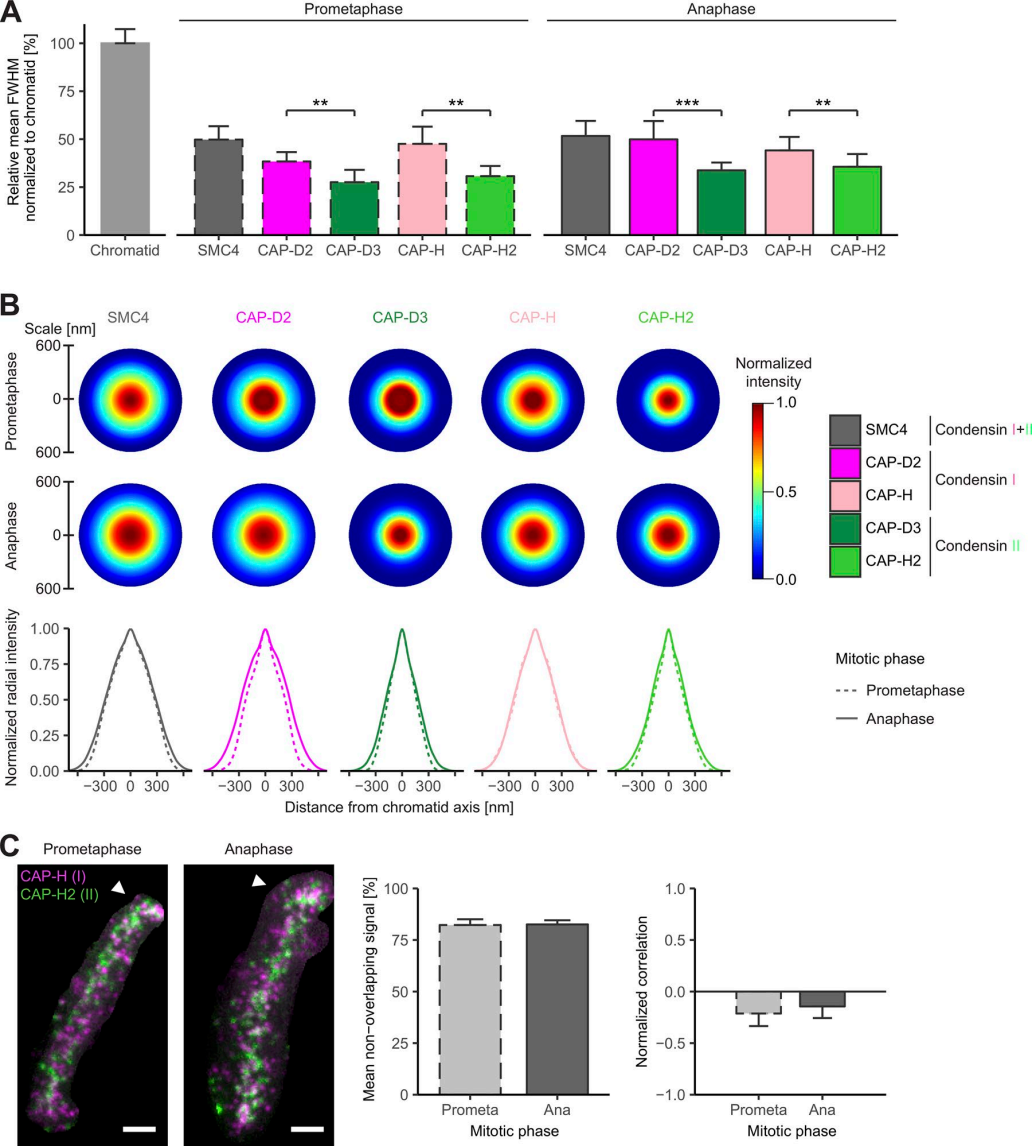
**Nonoverlapping localization of Condensins I and II along the mitotic chromatid axis. (A)** The FWHM of Condensin subunit distribution and chromatid arms was analyzed (for details, see Fig. S3, A and B). Condensin subunit FWHMs were plotted relative to the chromatid FWHMs in two mitotic phases (means + SD; ∼13 chromatids; *n* = 5–20; prometaphase: *n* = 7, 12, 5, 13, and 20 chromatids for CAP-H, CAP-H2, CAP-D2, CAP-D3, and SMC4, respectively; anaphase: *n* = 15, 16, 11, 20, and 15 chromatids for CAP-H, CAP-H2, CAP-D2, CAP-D3, and SMC4, respectively): prometaphase (dashed lines, left) and anaphase (solid lines, right). Student’s two-tailed *t* test: in prometaphase, P = 0.0034 for CAP-D2/CAP-D3, and P = 0.0014 for CAP-H/CAP-H2; in anaphase, P = 0.0002 for CAP-D2/CAP-D3, and P = 0.0016 for CAP-H/CAP-H2. Comparison of FWHM for the same Condensin subunit between prometaphase and anaphase: CAP-D2 (P = 0.0067), CAP-D3 (P = 0.0064), CAP-H (P = 0.3966), and CAP-H2 (P = 0.0391). **, P < 0.01; ***, P < 0.001. Because the chromatid FWHMs between prometaphase and anaphase were not significantly different, all chromatids were pooled for all subunits and mitotic phases (∼1.1 µm; *n* = 134). **(B)** Radial intensity distribution of Condensin subunits on chromatid arms (for details, see Fig. S3 C). xy Condensin intensity profiles were symmetrized, reconstructed as radially symmetric distribution, and centrally aligned with the chromatid. Condensin intensities were peak normalized per subunit and mitotic phase and are displayed within circles representing the chromatid width determined in A as diameter. Circular radial intensity profiles are shown for all analyzed Condensin subunits in prometaphase and anaphase (top). The z-projected (sum) Condensin intensity profiles are plotted for prometaphase (dashed lines) and anaphase (solid lines) together for each subunit (bottom). Averaged intensity profiles among all chromatids per subunit and mitotic stage are plotted (∼13 chromatids; *n* = 5–20). **(C)** Immunostaining of double-homozygously Condensin-tagged cell lines: Condensin I (CAP-H–Halo; magenta) and II (CAP-H2–mEGFP; green). One z plane of a representative prometaphase and anaphase chromatid is shown, and telomeres are indicated by white arrowheads (left). Bars, 500 nm. Although the labeling efficiencies of the two tags make a quantitative comparison of the protein abundances difficult, they were sufficiently high to allow us to test whether the two isoforms colocalize at the same chromosomal loci. Thus, Condensin I and II signals were segmented and analyzed for colocalization. Mean percentages of nonoverlapping segmented voxels between Condensins I and II are plotted (82% in both prometaphase and anaphase; middle). Normalized correlation of Condensin I and II signals is plotted (anticorrelated in both prometaphase and anaphase; right). Means + SD are shown (prometaphase, *n* = 19; anaphase, *n* = 18).

To quantify how far away from the chromatid center Condensins are localized, we used computational image analysis to determine the mean full width at half maximum (FWHM) of Condensin subunits relative to DNA (Fig. S3, A and B; for details, see Materials and methods). Condensin II was significantly more restricted to the center of the chromatid, occupying ∼30–35% of the ∼1,100-nm diameter of the chromatid arm (Fig. 3 A). In contrast, Condensin I localization could be detected at up to 50% of the chromatid diameter in both prometaphase and anaphase (Fig. 3 A). Interestingly, three out of four isoform-specific subunits displayed a statistically significant increase (∼100 nm) in their FWHM from prometaphase to anaphase, consistent with the idea that the final chromatid arm compaction (Mora-Bermúdez et al., 2007) results in a widening of the internal chromosome scaffold.

To determine where most of the Condensin molecules are located within the chromatid width, we quantified their radial intensity distributions perpendicular to the longitudinal chromatid axis (Fig. S3 C; for details, see Materials and methods). Consistent with the FWHM measurements, Condensin I subunits displayed a broader intensity distribution than Condensin II both in prometaphase and anaphase (Fig. 3 B). 90% of the Condensin I (CAP-D2) signal was contained within a diameter of 560 nm in prometaphase, extending to 760 nm in anaphase, whereas 90% of Condensin II (CAP-D3) was confined closer to the chromatid axis (480-nm and 560-nm diameters in prometaphase and anaphase, respectively). Interestingly, the shape of the radial intensity profile also differed between the isoforms. Condensin II subunits showed a narrow peak at the chromatid axis, consistent with an enrichment in the chromatid center, whereas Condensin I subunits showed a more bell-shaped profile with wider shoulders, consistent with the conclusion that many Condensin I complexes localize further away from the central chromatid axis (Fig. 3 B). This bell-like distribution of Condensin I broadened from prometaphase to anaphase (Fig. 3 B), indicating that most of the Condensin I mass that is added in anaphase binds distally from the central chromatid axis.

### Condensins I and II are not colocalized on mitotic chromatid arms

The differences in mean axial distribution of Condensins I and II suggest that the two complexes at least partially act at different loci on the chromosomal DNA molecule. To test this hypothesis directly, we imaged Condensins I and II in the same cell in 3D by two-color STED microscopy, which has a lateral resolution on the same scale as expected for the length of single Condensin complexes (Anderson et al., 2002). To this end, we generated a double-homozygous knock-in cell line in which we tagged CAP-H and CAP-H2 differentially with a Halo and an mEGFP tag. Remarkably, coimaging both kleisin subunits showed that Condensin I and II signals were anticorrelated, with ∼82% of the spot-like signals showing no spatial overlap in prometaphase or anaphase (Fig. 3 C; for details, see Materials and methods). The few colocalization events that we detected were enriched at centromeres and telomeres, where Condensins reach very high concentrations (Fig. 3 C, arrowheads), consistent with previous studies based on diffraction-limited imaging (Ono et al., 2004; Oliveira et al., 2005; Gerlich et al., 2006; Ribeiro et al., 2009). Interestingly, our superresolved images indicated an alternating localization of Condensins I and II along the chromatid arm, in line with previous studies that could not resolve single complexes (Maeshima and Laemmli, 2003; Ono et al., 2003). However, our higher-resolution data do not show quantifiable regular or periodic patterns.

In summary, our superresolution analysis of mitotic chromosomes in whole human cells shows that Condensins I and II differ in their localization within the chromatid, with Condensin II being confined to the axis and Condensin I binding more peripherally, as also shown for in vitro reconstituted chromatids (Shintomi et al., 2017), and that they do not bind to the same sites on mitotic chromatid arms.

### Genomic and physical spacing of Condensin subunits on mitotic chromosomes

**Figure 4.**
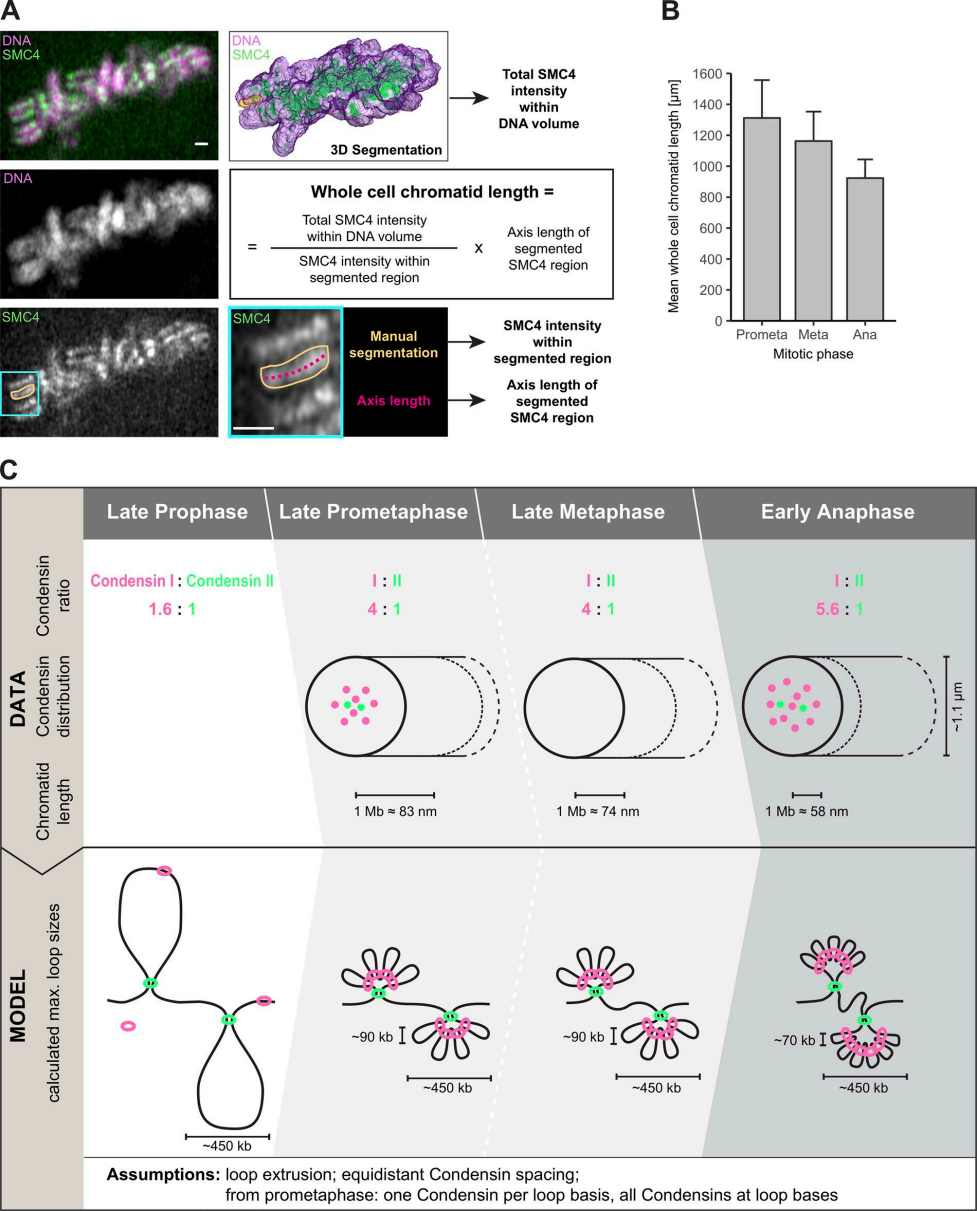
**A three-step hierarchical looping model of mitotic chromosome compaction based on a comprehensive 3D map of Condensins in human mitotic cells. (A and B)** Whole chromatid length in mitotic HK cells. **(A)** High-resolution z stacks of SMC4-tagged live cells stained with SiR-DNA to visualize chromosomes were acquired. For each cell, the total Condensin (green) and chromosome (magenta) volume was segmented in 3D, and the total Condensin intensity within the chromosome volume was determined. In addition, for each cell, several (three to five) clearly recognizable stretches of Condensin signal along the axis of chromatid arms were segmented manually, and their Condensin intensities and corresponding axis lengths were determined. These were used to compute the mean chromatid length per cell according to the formula indicated (see also Materials and methods). Bars, 1 µm. **(B)** Whole-cell chromatid lengths for late prometaphase, metaphase, and early anaphase (means + SD; *n* = 3 cells). **(C)** The quantitative 3D map of Condensins in human mitotic HK cells suggests a Condensin-based three-step hierarchical looping model of mitotic chromosome compaction. Step 1: Condensin II, which is present in the nucleus in interphase, promotes axial compaction in prophase. This compaction results in maximum ∼450-kb-long loops if fully extruded, with Condensin II localizing within the central 30–35% of the mitotic chromatid diameter (∼1.1 µm). Step 2: After NEBD in prometaphase, the majority of the in-interphase and prophase mainly cytoplasmic Condensin I gains access to mitotic chromosomes and binds to the central chromatid axis but also reaches more peripheral sites up to 50% of the chromatid diameter. The first phase of Condensin I binding leads to a fourfold higher abundance compared with Condensin II on metaphase chromosomes and drives mainly lateral compaction in prometaphase and metaphase by subdividing the large Condensin II loops four times into subloops of a size of ∼90 kb. In this step, the chromatid axis length constraining 1 Mb DNA decreases to 83 nm in prometaphase and further to 74 nm in metaphase. This Condensin I–driven subloop formation could in principle start already before NEBD based on the minor Condensin I fraction present on prophase chromosomes. Step 3: Upon anaphase onset, a second wave of Condensin I binds to mitotic chromosomes, reaching a 5.6-fold higher abundance than Condensin II and localizing even further away from the chromatid axis. These additional Condensin I complexes drive the final axial shortening step by further subdividing Condensin II loops into five to six subloops, leading to a final loop size of ∼70 kb, with 1 Mb DNA being now constrained to a 58-nm chromatid axis length when maximal chromosome compaction is reached at the end of anaphase. The Condensin abundances and spatial dimensions in the top half of the model were determined in this study (Condensin I/Condensin II ratio, chromatid width occupied by Condensin I/II, and relation between genomic and physical genome length). The loop sizes in the bottom half of the model calculated from these data are based on an equidistant Condensin spacing along the genome as well as the assumption of fully extruded loops according to the loop-extrusion theory whereby each loop base would be occupied by one Condensin holocomplex, and all Condensin holocomplexes are at loop bases (for prophase only for Condensin II and from prometaphase to anaphase for both Condensin complexes). However, the DNA path and spatial arrangement of loops in the bottom half of the model is drawn hypothetically to represent the determined DNA length.

To use our quantitative data of Condensin abundance, binding, and subchromosomal position to formulate a model for how chromosomal DNA molecules might be structured and compacted in mitosis, we needed to establish the relationship between physical distances and genomic lengths for mitotic chromosomes. Although the mean genome size of the HK genome is 7.9 billion bp (on an average hypotriploid, e.g., ∼64 chromosomes; Landry et al., 2013), its physical dimensions have not yet been determined. We therefore measured the total lengths of all chromatid axes per HK cell in different mitotic stages by tracing the SMC4-mEGFP–labeled chromatid axis relative to DNA in high-resolution 3D datasets collected from live cells (Fig. 4 A; for details, see Materials and methods). This approach avoids any fixation artifacts and thus enables direct comparison to our live-cell abundance measurements. Consistent with an increase in chromatid compaction during mitosis (Mora-Bermúdez et al., 2007; Hériché et al., 2014), the total chromatid length decreased from ∼1,300 µm in prometaphase to ∼1,150 µm in metaphase, reaching ∼925 µm in the most compacted state in anaphase (Fig. 4 B). Thus, an average HK chromatid of 123 Mb has a physical length of ∼9 µm in metaphase and contains ∼1,100 Condensin I (CAP-H) and ∼270 Condensin II (CAP-H2) holocomplexes (Table 1). During sister chromatid segregation in anaphase, the number of Condensin I holocomplexes increased to ∼1,500, and the chromatid was shortened to ∼7 µm (Table 1).

**Table 1.** Genomic and physical DNA length of an average HK cell, the absolute amount of Condensin subunits on mitotic chromosomes, and their linear spacing along the chromatid axis in late prometaphase, metaphase, and early anaphase

**Mitotic phase/parameter**	**Genomic length**	**Physical length**	**Number of SMC4 (I + II)**	**Number of CAP-D2 (I)**	**Number of CAP-H (I)**	**Number of CAP-D3 (II)**	**Number of CAP-H2 (II)**	**Number of CAP-H (I) + CAP-H2 (II)**
**Prometaphase**								
Total	2× 7.9 Gb	1,311.7 ± 245.2 µm	247,952 ± 43,519	152,558 ± 20,965	145,478 ± 27,626	73,267 ± 14,861	36,451 ± 13,604	~181,929
Chromatid	123.4 Mb	~10.2 µm	~1,937	~1,192	~1,137	~572	~285	~1,421
1 µm	~12.0 Mb	1.0 µm	~189	~116	~111	~56	~28	~139
1 Mb	1.0 Mb	~83.0 nm	~16	~10	~9	~5	~2	~12
Genomic Condensin spacing			~63.7 kb	~103.6 kb	~108.6 kb	~215.6 kb	~433.5 kb	~86.8 kb
Physical Condensin spacing			~5.3 nm	~8.6 nm	~9.0 nm	~17.9 nm	~36.0 nm	~7.2 nm
**Metaphase**								
Total	2× 7.9 Gb	1,162.5 ± 190.4 µm	252,652 ± 39,130	153,519 ± 22,952	140,965 ± 26,521	71,408 ± 14,999	34,543 ± 12,521	~175,509
Chromatid	123.4 Mb	~9.1 µm	~1,974	~1,199	~1,101	~558	~270	~1,371
1 µm	~13.6 Mb	1.0 µm	~217	~132	~121	~61	~30	~151
1 Mb	1.0 Mb	~73.6 nm	~16	~10	~9	~5	~2	~11
Genomic Condensin spacing			~62.5 kb	~102.9 kb	~112.1 kb	~221.3 kb	~457.4 kb	~90.0 kb
Physical Condensin spacing			~4.6 nm	~7.6 nm	~8.2 nm	~16.3 nm	~33.7 nm	~6.6 nm
**Anaphase**								
Total	2× 7.9 Gb	923.1 ± 120.7 µm	325,522 ± 48,878	216,857 ± 32,900	194,427 ± 37,121	71,244 ± 16,174	34,677 ± 13,294	~229,104
Chromatid	123.4 Mb	~7.2 µm	~2,543	~1,694	~1,519	~557	~271	~1,790
1 µm	~17.1 Mb	1.0 µm	~353	~235	~211	~77	~38	~248
1 Mb	1.0 Mb	~58.4 nm	~21	~14	~12	~5	~2	~15
Genomic Condensin spacing			~48.5 kb	~72.9 kb	~81.3 kb	~221.8 kb	~455.6 kb	~69.0 kb
Physical Condensin spacing			~2.8 nm	~4.3 nm	~4.7 nm	~13.0 nm	~26.6 nm	~4.0 nm

Calculations are based on the 7.9-billion-bp HK genome with on average 64 chromosomes (Landry et al., 2013), the whole-cell chromatid lengths for late prometaphase, metaphase, and early anaphase (Fig. 4 B), and the numbers of Condensin subunits bound to chromosomes as determined by FCS-calibrated imaging (Fig. 1, C and D). The genomic and physical Condensin spacing assumes an equidistant distribution of Condensins along the mitotic chromatid axis.

Given the absence of periodic patterns or regular clusters along the chromatid axis, we assume for simplicity an equidistant spacing of Condensins along chromatid arms to predict the genomic spacing of the ∼1,400 Condensin holocomplexes on an average prometaphase HeLa chromatid. This calculation resulted in a mean genomic distance between neighboring Condensins of ∼90 kb in prometaphase and metaphase, which would be reduced to ∼70 kb by the binding of additional Condensin I complexes in anaphase (Table 1). If we further assume that each Condensin holocomplex defines a DNA loop, Condensins would be able to form ∼90-kb loops on prometaphase chromosomes, which would be spaced ∼7 nm apart along the chromatid axis (123 Mb ≈ 10.2 µm, and 90 kb ≈ 7 nm), with most loops bound by Condensin I and only every fifth loop by Condensin II. This makes the clear prediction that Condensin II complexes should on average be spaced ∼35 nm apart along the chromatid axis.

Because STED superresolution imaging in principle has a resolving power of ∼30 nm in 2D, we analyzed our STED data of prometaphase chromosomes for the limiting kleisin subunit of Condensin II (CAP-H2) to see whether the number of detected localizations and their spatial distribution matched these predictions. Using 3D computational image analysis (Fig. S3 D; for details, see Materials and methods), we could detect 16 spot-like localizations of Condensin II per µm of chromatid axis, slightly more than half of the 28 predicted from the abundance measurement by FCS-calibrated imaging. This indicates a combined labeling and detection efficiency of ∼60% by the combination of our primary and secondary antibody (AB) bearing the STED dye, making it very likely that most localizations represent single Condensin II complexes. We observed a median axial spacing of 56 nm (if projected onto the chromatid axis; Fig. S3, D and G; for details, see Materials and methods). Assuming that we label and detect ∼60% of the complexes present, this number is in very good agreement with our predicted axial spacing of 35 nm from FCS-calibrated imaging. In anaphase, the observed median axial spacing decreased from 56 to 50 nm, whereas the distance from the axis increased from 115 to 133 nm, consistent with the mean intensity and FWHM measurements (Fig. 3, A and B).

### Data-driven three-step hierarchical loop model of mitotic chromosome compaction

Our integrated quantitative imaging data provide the absolute abundance, physical spacing, and dynamic binding of Condensins on mitotic chromosomes. Together with the calibration of physical and genomic length of the mitotic genome in a human cell, these parameters allowed us to estimate the maximum sizes of DNA loops that could be formed by Condensins to structure mitotic chromosomes. Taking into account the prevailing model of the loop-extruding and stabilizing function of Condensin complexes (Nasmyth, 2001; Alipour and Marko, 2012; Goloborodko et al., 2016a,b), we therefore propose a three-step hierarchical loop model for how Condensins compact mitotic chromosomes (Fig. 4 C). Condensin II is nuclear during interphase, and its abundance on chromosomes is constant throughout mitosis (Fig. 1, A and C). It likely promotes the initial axial compaction step in prophase and achieves this within the central 30–35% of the chromatid diameter (Fig. 3, A and B). Based on the measured number as well as the measured physical and calculated genomic spacing of Condensin II complexes (Table 1), these loops would be large and ∼450 kb in size if fully extruded. In contrast, the majority of Condensin I only gains access to mitotic chromosomes upon NEBD in prometaphase (Fig. 1, A and C), when it binds to the central chromatid axis but also reaches more peripheral sites, occupying up to 50% of the chromatid diameter (Fig. 3, A and B). The first phase of Condensin I binding leads to a fourfold higher abundance compared with Condensin II on metaphase chromosomes (Fig. 1, C and D) and likely drives lateral compaction in prometaphase and metaphase by subdividing the large Condensin II–mediated loops four times into subloops of sizes of ∼90 kb. Upon anaphase onset, a second wave of Condensin I binds to mitotic chromosomes (Fig. 1, A and C), which then reaches a 5.6-fold higher abundance than Condensin II (Fig. 1, C and D) and localizes even further away from the chromatid axis (Fig. 3, A and B). These additional Condensin I complexes might drive the final axial shortening step by further subdividing the large Condensin II loops into five to six subloops, leading to a final mean loop size of ∼70 kb. The short (∼2 min) residence time of Condensin I as determined by FRAP (Fig. S2 K) would allow such dynamic changes in subloop architecture to occur on mitotically relevant time scales.

Our proposed model for the Condensin-based formation and structural organization of mitotic chromosomes is based on the following parameters determined in this study and assumptions supported by the current literature (Nasmyth, 2001; Alipour and Marko, 2012; Goloborodko et al., 2016a,b). Condensin holocomplexes are defined by the number of limiting kleisin subunits, which we determined by FCS; these were on average equidistantly spaced along the genome based on our superresolution localizations. All Condensin holocomplexes define loop bases, with one Condensin per DNA loop as supported by a recent study (Ganji et al., 2018). The endpoint states of each mitotic phase illustrated in our model thus represent the maximum size of fully extruded Condensin-mediated loops under these assumptions (Fig. 4 C). Using this logical and quantitative framework allowed us to integrate our findings of two consecutive Condensin I recruitment waves (Fig. 1 C), the higher abundance of chromosome-bound Condensin I (Fig. 1, C and D) as well as the larger distance of Condensin I from the chromatid center (Fig. 3, A and B) with the nonoverlapping localization of the two Condensin complexes (Fig. 3 C), and to propose a comprehensive model of Condensin II forming large prophase loops and Condensin I shortening these loops by the generation of smaller subloops in prometaphase and anaphase onset.

Because the direct visualization and size determination of single DNA loops in whole cells remains to our knowledge a technically unsolved challenge, our postulated loop arrangement based on our quantitative and superresolved data is speculative and intended to generate testable hypotheses to further advance the field. We note that our imaging-based model for human cells is consistent with a biochemical study of drug-synchronized chicken cells published during the revision of this work, which proposed a conceptually similar arrangement of large Condensin II–based and smaller Condensin I–based loops (Gibcus et al., 2018). This orthogonal chromosome conformation capture (Hi-C) study furthermore computationally predicted a helical arrangement of Condensin II along the mitotic chromosome axis and provided a few diffraction-limited images of Condensin II localization of hypotonically spread chromosomes. Although our model did not predict a helical arrangement and it is not evident by manual inspection of our data (e.g., Fig. 3 C), the Hi-C study prompted us to examine our extensive superresolved dataset of Condensin II localization from whole cells by unbiased computational image analysis and statistical data mining. This analysis did not detect significant helical or otherwise periodic patterns of Condensin II along the chromatid axis, which can therefore neither be supported nor excluded by our data.

Importantly, our quantitative imaging data-driven model is consistent with data derived by orthogonal biochemical cross-linking approaches that have proposed similar loop sizes in mitotic human HeLa (Naumova et al., 2013) or HAP1 cells (Elbatsh et al., 2017) as well as chicken cells (Gibcus et al., 2018). Our single-cell imaging–based results, however, provide for the first time absolute quantitative parameters regarding the abundance, physical spacing relative to chromatid geometry, and genomic spacing of both human Condensin complexes. This will be a valuable resource for future models of mitotic chromosome structure and compaction. One way to validate our model further in the future would be to directly resolve single Condensins on the individual loops of the DNA molecule of a mitotic chromatid. This will require new labeling and imaging methods for DNA and proteins that can provide isotropic nanometer-scale resolution in all three dimensions as well as an imaging depth sufficient to penetrate deep into mitotic cells.

## Materials and methods

### Cell culture

HK cells were obtained from S. Narumiya (Kyoto University, Kyoto, Japan) and grown in 1× high-glucose DMEM (41965039; Thermo Fisher Scientific) supplemented with 10% (vol/vol) FBS (10270106; Thermo Fisher Scientific; qualified, European Union approved, and South American origin), 100 U/ml penicillin-streptomycin (15140122; Thermo Fisher Scientific), 2 mM l-glutamine (25030081; Thermo Fisher Scientific), and 1 mM sodium pyruvate (11360070; Thermo Fisher Scientific) at 37°C and 5% CO_2_ in cell culture dishes (Thermo Fisher Scientific) in a cell culture incubator. Cells were passaged every 2–3 d by trypsinization (Trypsin-EDTA [0.05%] and phenol red [25300054; Thermo Fisher Scientific]) at a confluency of 80–90%. Cells were confirmed to be mycoplasma free on a regular basis.

### Cell line generation

HK cells were used for genome editing to express endogenously tagged proteins of interest (POIs). For tagging SMC4 at the C terminus and CAP-H at the N terminus with mEGFP, zinc finger nucleases (ZFNs) containing DNA binding sequences listed in Table S1 were purchased from Sigma-Aldrich. The donor plasmids consisted of the mEGFP cDNA sequence flanked by left and right homology arms listed in Table S2. ZFN pairs and the donor plasmids were transfected into HK cells as described by Mahen et al. (2014) and Koch et al. (2018). For tagging CAP-D2 and CAP-D3 (Wutz et al., 2017) at the C terminus, CAP-H and CAP-H2 at the C terminus, and CAP-H2 at the N terminus (Cai et al., 2017) with mEGFP, paired CRISPR/Cas9D10A nickases were used. The design of guide RNAs (gRNAs) was performed as described by Otsuka et al. (2016) and Koch et al. (2018) (gRNAs listed in Table S1) and transfected together with donor plasmids (donor plasmids listed in Table S2) as described by Otsuka et al. (2016) and Koch et al. (2018). For SMC4 and CAP-D2, only heterozygous clones were achieved after the first round of genome editing. Thus, after confirming by Sanger sequencing that the nontagged alleles were not mutated, a second round of genome editing identical to the first round was performed on top of a heterozygous clone, resulting in homozygous clones. The double-endogenously tagged cell line HK CAP-H2–mEGFP CAP-H–Halo was generated by using the homozygous HK CAP-H2–mEGFP clone as a parental cell line and performing genome editing based on the paired CRISPR/Cas9 D10A nickase approach (gRNAs listed in Table S1; donor plasmids listed in Table S2). All genome-edited cell lines were confirmed to be mycoplasma free on a regular basis. The tagging strategy was designed so that all Condensin isoforms could be tagged in the C-terminally mEGFP-tagged SMC4, CAP-D2, CAP-D3, and CAP-H cell lines. For CAP-H2, one theoretically predicted but not experimentally detected isoform could not be tagged in the C-terminally mEGFP-tagged cell line, but all isoforms could be tagged in the N-terminally mEGFP-tagged cell line. FCS-calibrated imaging of metaphase cells did not reveal a significant difference between the abundance of CAP-H2 proteins on metaphase chromosomes between N- and C-terminally tagged clones (see Fig. S2 G; two-sided Kolmogorov-Smirnov test; P = 0.1732 comparing data from eight C-terminally tagged clones, *n* = 81 cells, and five N-terminally tagged clones, *n* = 41 cells). Thus, C-terminally mEGFP-tagged clones for all Condensin subunits were used throughout this study apart from Fig. S2 (F and G), which shows data from both C- and N-terminally tagged CAP-H and CAP-H2 clones, respectively.

### Cell line validation

#### Junction PCR, Southern blotting (SB), Western blotting (WB), confocal microscopy, mitotic timing analysis, and Sanger sequencing

All single- and double-endogenously tagged cell lines were validated as described by Mahen et al. (2014), Otsuka et al. (2016), and Koch et al. (2018) by performing junction PCRs to test for integration and homozygosity (primers listed in Table S3), SB analysis with endogenous and GFP probes (and a Halo probe for double-tagged cell lines) to test for homozygosity, local genome rearrangements and additional integrations of the GFP or Halo donors (probes listed in Table S5), WB analysis to test for the expression of homozygously tagged proteins and absence of free GFP expression (ABs listed in Table S4), confocal microscopy to test for correct localization of the tagged proteins, widefield time-lapse microscopy and automated classification into mitotic phases (Held et al., 2010) to confirm that mitotic timing (from prophase to anaphase onset) was not perturbed, and Sanger sequencing. To confirm the correct localization of Halo-tagged CAP-H in the double-endogenously tagged cell line, cells were stained with 100 nM Halo Tag TMR ligand (G8251; Promega) in prewarmed complete medium for 10 min at 37°C and 5% CO_2_ and washed three times with prewarmed 1× PBS (homemade) followed by changing to imaging medium (CO_2_-independent imaging medium [custom order based on 18045070 from Thermo Fisher Scientific; without phenol red] supplemented with 20% [vol/vol] FBS [10270106; Thermo Fisher Scientific], 2 mM l-glutamine [25030081; Thermo Fisher Scientific], and 1 mM sodium pyruvate [11360070; Thermo Fisher Scientific]) and then incubated for 30 min at 37°C in the microscope incubation chamber before confocal microscopy as described by Koch et al. (2018). In addition, coimmunoprecipitation against the GFP tag was performed for all selected single-homozygously mEGFP-tagged cell lines (HK 2× ZFN SMC4-mEGFP 82_68, HK 2× CRISPR CAP-D2–mEGFP 272_78, HK CRISPR CAP-D3–mEGFP 16, HK ZFN mEGFP–CAP-H 9, HK CRISPR CAP-H–mEGFP 86, HK CRISPR mEGFP–CAP-H2 1, HK CRISPR CAP-H2–mEGFP 67, and HK CRISPR CAP-H2–mEGFP 67 CRISPR CAP-H–Halo 29) to confirm the assembly of Condensin subunits into pentameric complexes (ABs listed in Table S4; for details, see next section).

#### Anti-GFP IP and WB against Condensin subunits

GFP–Trap A beads (gta-20; ChromoTek) were used to pull down mEGFP-tagged proteins in HK cell extracts. Cells grown to 80–90% confluence were harvested either as an asynchronously cycling population or after 18 h incubation with 330 nM nocodazole (Noc; 48792; Merck) to yield a mitotically arrested cell population. Cell pellets were lysed in the following lysis buffer: 10% glycerol (104093; Merck), 1 mM DTT (D-9779; Sigma-Aldrich), 0.15 mM EDTA (324503; Merck), 0.5% Triton X-100 (T8787; Sigma-Aldrich), one tablet of cOmplete EDTA protease inhibitor (11873580001; Roche), and one tablet PhosSTOP (04906837001; Roche). Lysis was performed on ice for 30 min, and the suspension was centrifuged at 13,000 rpm and 4°C for 10 min. The protein concentrations of the cell extracts were determined by protein assay (500-0006; Bio-Rad Laboratories), and 400 µg of total protein was used for IP. GFP–Trap A beads were washed twice with 1× PBS (homemade) and used at 1:1 dilution in PBS. 400 µg of total protein extracts were diluted 1:1 with PBS and incubated with the diluted GFP–Trap A beads at 4°C for 1 h. After the incubation, SN samples were taken, and the beads were washed twice with lysis buffer at 1:1 dilution with PBS. After two additional washing steps with PBS, 20 µl 4× NuPAGE LDS sample buffer (NP0008; Thermo Fisher Scientific) supplemented with 100 µM DTT was added to the beads and incubated at 65°C for 5 min to elute the protein from the beads. Input (IN), SN, and eluate (IP) samples were taken for WB against tagged and untagged subunits of the Condensin complexes (ABs listed in Table S4).

### FCS-calibrated confocal time-lapse imaging (sample preparation, FCS-calibrated confocal time-lapse imaging, data processing, and analysis)

Samples for FCS-calibrated confocal time-lapse imaging were prepared as described by Cai et al. (2017) and Politi et al. (2018). In brief, 2 × 10^4^ cells that had been passaged the day before were seeded into individual chambers of a Nunc eight-well LabTek chambered coverglass (1.0; 155411; Thermo Fisher Scientific) and incubated overnight (ON) at 37°C and 5% CO_2_ in a cell culture incubator. 2 h before imaging, the medium was changed to imaging medium (see the first subsection of Cell line validation) containing 50 nM SiR-DNA (SC007; Spirochrome; Lukinavičius et al., 2015) and 3.3 µM of 500-kD dextran (D7144; Thermo Fisher Scientific) labeled with Dy481XL (481XL-00; Dyomics; Dextran-Dy481XL was produced in house).

FCS-calibrated confocal time-lapse imaging was performed as described by Cai et al. (2017) and Politi et al. (2018) (Fig. S1, D–H). FCS measurements of a 50-nM fluorescent dye solution of Alexa Fluor 488 (A20000; Thermo Fisher Scientific) in double-distilled H_2_O were performed to estimate the confocal volume. FCS measurements in both the nucleus and the cytoplasm of WT cells not expressing mEGFP were performed to determine background fluorescence and background photon counts. FCS measurements in both the nucleus and the cytoplasm of WT interphase cells expressing free mEGFP as well as interphase cells homozygously expressing an mEGFP-tagged Condensin subunit were performed to estimate an experiment-specific calibration factor that was used to transform mEGFP fluorescence into mEGFP concentration (Fig. S1 G). Prophase cells expressing an mEGFP-tagged Condensin subunit were automatically detected in low-resolution imaging mode based on the DNA staining (SiR-DNA) using CellCognition (Held et al., 2010). Upon recognition of a prophase cell, high-resolution time-lapse imaging of this cell through mitosis was triggered. Hereby, a z stack covering the whole volume of the cell was acquired every 1.5 min for a total of 60 min in the mEGFP (mEGFP-tagged Condensin subunit), SiR-DNA (DNA), Dextran-Dy481XL (regions outside cells), and transmission channels. After each mitotic time lapse, one of the two daughter nuclei was detected, and six FCS measurements were acquired, three each inside and outside the nucleus, and used in addition to the interphase measurements for establishing the FCS calibration curve to transform mEGFP fluorescence intensities in each z stack of a mitotic time-lapse video into protein concentrations. A 3D segmentation pipeline was used to detect and track cells through mitosis as well as to reconstruct chromosomal and cell surfaces from the SiR-DNA and Dextran-Dy481XL channels, respectively (Cai et al., 2017). To compensate for the variation of mitotic progression between individual cells, a mitotic standard time was computed based on geometric features of chromosomes, and each cell was aligned to this mitotic standard time of 60 min (Cai et al., 2017). FCS data processing, generation of calibrated images, and data analysis to create cellular protein concentration and number maps (for details, please refer to the Estimation of protein numbers from FCS-calibrated images section) including a multistep quality control pipeline were performed as described by Wachsmuth et al. (2015), Cai et al. (2017), and Politi et al. (2018).

### Estimation of protein numbers from FCS-calibrated images

To estimate the number of proteins, fluorescent images of dividing cells were processed according to Cai et al. (2017) and Politi et al. (2018). Protein fluorescence intensities in image voxels were converted into numbers of proteins using the FCS calibration curve (Fig. S1, G and H). 3D segmentations of the Dextran-DY481XL and SiR-DNA channels defined binary masks for the cell and chromatin/nuclear regions, respectively (Fig. S1 I). The region in the cell but not in the chromatin region (XOR operation) was used to specify the cytoplasmic region. The total number of proteins was computed by adding up all proteins within the cell mask. Mean concentrations in the chromatin/nucleus [*Chr*] and cytoplasm [*Cyt*] regions were computed by dividing the total number of proteins in the corresponding region by its volume. The FRAP results indicate that Condensin subunits can freely diffuse within the chromatin region (Fig. S2, H–L). To compute the number of proteins bound to chromatin, we subtracted this freely diffusible protein pool. Between NEBD (mitotic standard stage 4) and the end of anaphase (mitotic standard stage 14; Cai et al., 2017), the nuclear envelope is not sealed. Thus, the concentration of the freely diffusible protein pool could be assumed to be equal to the measured cytoplasmic concentration [*Chr*]*_bg_* = [*Cyt*]. This yielded the following corrected concentration and total number of chromatin-bound proteins:

$${\left[Chr\right]}_{c}=\left(\left[Chr\right]-\left[Cyt\right]\right)$$ (1)

and

$$N_{Chrc}={\left[Chr\right]}_{c}\;V_{Chr}\;N_{A},$$ (2)

as well as a corrected total number of proteins in the cytoplasm:

$$N_{Cyt}=\left(\left[Cyt\right]\;V_{Cyt}+\left[Cyt\right]V_{Chr}\right)\;N_{A},$$ (3)

where *V_Chr_* and *V_Cyt_* are the volumes of the chromatin region and cytoplasmic region, respectively, and *N_A_* is the Avogadro constant. Before NEBD (prophase), we assumed that binding and unbinding of the Condensin subunits to chromatin is in equilibrium. The equilibrium constant *K_d_* was estimated from the concentrations in metaphase:

$$K_{d}=\frac{\left[Cyt\right]\left(meta\right)}{{\left[Chr\right]}_{c}\left(meta\right)}.$$

Thus, the freely diffusible nuclear concentration before NEBD is

$${\left[Chr\right]}_{bg}=\frac{K_{d}}{1+K_{d}}\left[Chr\right]$$ (4)

and the corrected chromatin-bound concentration is

$${\left[Chr\right]}_{c}=\frac{\left[Chr\right]}{1+K_{d}}.$$ (5)

The number of proteins was calculated by multiplying these values with the chromatin volume. After nuclear envelope reformation, an equilibrium constant between chromatin-bound and free-diffusing Condensin subunits could not be determined. Therefore, we only computed the total number of proteins in the nucleus.

### FRAP experiments and analysis

Cells were seeded as for FCS-calibrated imaging. 2 h before imaging, the medium was changed to imaging medium (see the first subsection of Cell line validation) containing 50 nM SiR-DNA to stain the DNA. Experiments were performed on an LSM780 laser-scanning microscope with an inverted Axio Observer operated by the ZEN 2012 Black software (ZEISS). The microscope was equipped with a temperature-controlled incubation chamber (constructed in house), and the temperature was set to 37°C for imaging of living cells. Images were acquired using a C-Apochromat 40× 1.20 W Korr FCS M27 water-immersion objective (ZEISS) with an in house–built objective cap connected to an automated pump control system and spectral gallium arsenide phosphide (GaAsP) detectors (ZEISS). Cells in metaphase were manually selected based on their DNA staining and imaged every 20 s for 30–45 frames (nine z planes; xyz pixel size: 0.25 × 0.25 × 0.75 µm). Condensin-mEGFP was excited with 488 nm (argon laser at 2%) and detected with GaAsP detectors at 500–535 nm. SiR-DNA was excited with 633 nm (HeNe laser at 0.3%) and detected with GaAsP detectors at 642–695 nm. After one prebleach image, a region of interest (ROI) covering approximately half of the chromatin (metaphase plate) in the fifth z plane was bleached using a squared region of 40 × 30 pixels (488 nm laser at 100%; 100 repetitions), and time-lapse videos were recorded.

A custom-written ImageJ (National Institutes of Health; Schindelin et al., 2012) script was used to extract the mean fluorescence intensity of the mEGFP-tagged POI and SiR-DNA in the bleached and unbleached chromatin region. To this end, the SiR-DNA channel was segmented to obtain the chromatin mask. The mask was then applied to the SiR-DNA and mEGFP channels previously filtered with an anisotropic 2D diffusion filter (Tschumperlé and Deriche, 2005). For every time point and z plane, the 1D profile of the SiR-DNA and mEGFP fluorescence intensity along the major 2D axis of chromatin was computed. Data close to the center of the 1D profile, which corresponded to the border of the bleaching ROI, were omitted to avoid boundary effects. In this experiment, a gap of 14 pixels (3.5 µm) was used. The weighted mean of the fluorescence intensity of the POI was computed using the SiR-DNA intensities as weights. The mean fluorescence intensity in the bleached (*F_b_*) and unbleached (*F_ub_*) regions were obtained as follows:

$$\begin{array}{l} \;F_{b}\left(t\right)=\frac{\sum_{x}P_{b}\left(x,t\right)*D_{b}\left(x,t\right)}{\sum_{x}D_{b}\left(x,t\right)},\;\\ F_{ub}\left(t\right)=\frac{\sum_{x}P_{ub}\left(x,t\right)*D_{ub}\left(x,t\right)}{\sum_{x}D_{ub}\left(x,t\right)}, \end{array}$$ (6)

where *P_b,ub_(x, t)* and *D_b,ub_(x, t)* are the POI and SiR-DNA fluorescence intensities at position *x* along the DNA axis and at time *t*, respectively. The normalized difference between the unbleached and bleached region:

$$\frac{F_{ub}\left(t\right)-\;F_{b}\left(t\right)}{F_{ub}\left(0\right)-\;F_{b}\left(0\right)}$$

was used as a readout for the residence time and immobile fraction (Gerlich et al., 2006).

Assuming a binding equilibrium of the POI with the chromatin and a constant total amount of protein, a system of differential equations for the unbleached and bleached regions was obtained. The solution of the differential equations gives a closed expression for the normalized difference between unbleached and bleached regions:

$$\frac{F_{ub}\left(t\right)-\;F_{b}\left(t\right)}{F_{ub}\left(0\right)-\;F_{b}\left(0\right)}=a+\left(1-a\right)\;e^{-\left(\kappa +k_{off}\right)\;t}.$$ (7)

In this equation, the forward rate constant *K* = *k_on_ B* is the product of the binding rate constant *k_on_* and the number of binding sites *B*, *k_off_* is the unbinding rate constant, and *a* is the immobile fraction. The benefit of using the difference between unbleached and bleached regions is that all terms describing photobleaching cancel out and do not appear in Eq. 7. The residence time is given by

$$\tau =\frac{1}{\kappa +k_{off}}$$ (8)

Different from classical point FRAP, where τ ≈ 1/*k_off_*, the residence time depends on the binding and unbinding rate constants. This is a consequence of the fact that a large portion of the proteins were bleached.

### Sample preparation for STED microscopy

To prepare HK cells homozygously expressing an mEGFP-tagged Condensin subunit for STED microscopy, 2.5 × 10^5^ cells that had been passaged the day before were seeded on squared glass coverslips (0107032; Marienfeld), prewashed with 1× PBS in Nunc six-well plates (140685; Thermo Fisher Scientific), and incubated ON at 37°C and 5% CO_2_ in a cell culture incubator. On the next day, the asynchronously cycling cell population was washed once with 1× PBS (+ Ca^2+^/Mg^2+^; 0.9 mM CaCl_2_ and 0.5 mM MgCl_2_; homemade). Preextraction was performed with 0.1% Triton X-100 (T8787; Sigma-Aldrich) in 1× PBS (+ Ca^2+^/Mg^2+^) for 3 min at RT to permeabilize cell and nuclear membranes and remove soluble proteins followed by two additional rounds of washing with 1× PBS (+ Ca^2+^/Mg^2+^). All steps up to this one were performed very carefully to not lose rounded up and thus easily detachable mitotic cells. Cells were fixed with 4% PFA (15710; Electron Microscopy Sciences) in 1× PBS (+ Ca^2+^/Mg^2+^) for 15–20 min at RT. After three washing steps with 1× PBS, samples were quenched by incubation in 50 mM Tris-Cl, pH 7–8 (homemade) for 5 min followed by two washes with 1× PBS for 5 min each. Further permeabilization was performed by incubation in 0.2% Triton X-100 in 1× PBS for 10 min followed by three brief washing steps and two 5-min washes in 1× PBS. Samples were blocked in 2% (wt/vol) BSA (A2153; Sigma-Aldrich) in 1× PBS for 1 h at RT. Incubation with the primary anti-GFP AB (polyclonal rabbit anti-GFP AB; 1:500; 598; MBL) in blocking solution (2% [wt/vol] BSA in 1× PBS) was performed ON at 4°C. Three 5-min washes with blocking solution were followed by incubation with the secondary AB coupled to a STED dye (1:500; polyclonal goat anti–rabbit Abberior STAR RED; 2–0012-011-9; Abberior) in blocking solution for 1 h at RT. Samples were washed once with 1× PBS and incubated with Hoechst 33342 (1:10,000; H3570; Thermo Fisher Scientific) in 1× PBS for 10 min at RT followed by three washes with 1× PBS for 5 min. Samples were mounted in DABCO-glycerol (1% [wt/vol] DABCO [D27802; Sigma-Aldrich] and 90% [vol/vol] glycerol [104093; Merck] in 1× PBS) on glass slides (12114682; Thermo Fisher Scientific), sealed with Picodent twinsil (13001000; Picodent), and kept at 4°C in the dark until imaging. STED imaging was performed as soon as possible after sample preparation.

To prepare HK CAP-H2–mEGFP CAP-H–Halo cells for STED imaging, the same protocol was used apart from the following changes: The polyclonal primary ABs anti-GFP (chicken anti-GFP; 1:500; ab13970; Abcam) and anti-Halo (rabbit anti-Halo; 1:500; G9281; Promega) as well as the polyclonal goat secondary ABs anti–chicken Alexa Fluor 594 (1;500; A-11042; Thermo Fisher Scientific) and anti–rabbit Abberior STAR RED were used in blocking solution. For primary AB staining, samples were preincubated with rabbit anti-Halo AB for 1 h at RT before ON incubation with rabbit anti-Halo AB and chicken anti-GFP AB at 4°C. The two secondary ABs were incubated together for 1 h at RT. No DNA staining with Hoechst was performed.

### STED imaging

STED imaging was performed on a combined Abberior STED and RESOLFT system (Expert line; Abberior) operated by Imspector software (v0.13.11885; Abberior). Samples were imaged with a UPlan-S Apochromat 100× 1.4 NA oil-immersion objective on an IX83 stand (Olympus). The microscope was equipped with an incubation chamber (constructed in house), and a temperature of 22.5 ± 0.2°C was ensured by constant cooling to minimize sample drift and optimize optical performance. For STED imaging, a 775-nm pulsed depletion laser (1.25 W total power; attenuated for actual imaging; acoustooptic tunable filter set to 40%; MPBC) was used, which created a doughnut-shaped 2D depletion beam, increasing the resolution laterally but not axially in comparison with a confocal microscope.

For diffraction-limited and STED imaging of mEGFP-tagged Condensin subunits stained with Abberior STAR RED, the dye was excited with a 640-nm pulse excitation laser, and emitted photons were recorded on an avalanche photodiode (APD) with a 650–720-nm band pass filter in line sequential mode. For STED imaging, the pulsed 640-nm excitation laser was triggered by the 775-nm pulsed STED depletion laser with 1-ns pulse length. A detection delay of 781.3 ps with respect to the excitation pulse was applied. In addition, DNA stained by Hoechst 33342 and the mEGFP-tagged Condensin subunit were imaged in diffraction-limited in-line sequential mode using 405-nm or 488-nm excitation lasers, respectively, and a 500–550-nm band pass filter for detection on a separate APD. The pinhole was set to 1.2 airy units, and the pixel dwell time was set to 2 µs.

For overview images of whole cells as shown in Fig. 2, a single z plane was acquired with the following settings: Eight line accumulations were performed, each of the following sequence: DNA (405-nm excitation laser, 10% [corresponding mean power of 2.1 µW at the objective]) and GFP (488-nm excitation laser, 25% [28.8 µW]) channels were accumulated three times, whereas both the diffraction-limited (640-nm excitation laser, 2% [4.0 µW]) and the STED (640-nm excitation laser, 2% [4.0 µW]; 775-nm depletion laser, 40% [166.3 mW]) channels of the immunostained Condensin subunit were accumulated five times, resulting in a total of 24 accumulations for diffraction-limited imaging of DNA and GFP as well as a total of 40 accumulations for diffraction-limited and STED imaging of the immunostained Condensin subunit. For acquiring z stacks used for the analyses in Fig. 3 (A and B) and Fig. S3, selected chromatids were imaged only in the DNA channel (diffraction limited) and the immunostained Condensin subunit (STED) channel to minimize bleaching by using otherwise the same settings as described for overview images.

Mitotic cells in late prometaphase and early anaphase with chromatids having their longitudinal axes oriented parallel to the focus plane were selected for imaging based on their DNA and Condensin-mEGFP signals. The pixel size in xy was 20 nm. Z stacks were acquired using the focus stabilizer/z drift control ZDC2 (custom-modified for STED use; Olympus) by focusing via the offset of the device and thereby ensuring precise axial positioning of each slice. To cover whole chromosomes in 3D, z stacks of 29 slices and a z interval of 140 nm were acquired. By this z depth of ∼4 µm, it could be ensured that the whole 3D volume of not only straight but also slightly bent chromosomes was covered.

0.1-µm TetraSpeck microspheres (T7279; Thermo Fisher Scientific) mounted on glass slides were imaged to align STED and excitation beams and to determine a potential offset between the diffraction-limited DNA and superresolved immunostained Condensin-mEGFP channels. If necessary, an offset was corrected before performing the analyses shown in Fig. 3 (A and B) and Fig. S3.

For two-color STED microscopy of Condensins I and II as shown in Fig. 3 C, chromatid regions of HK CAP-H2–mEGFP CAP-H–Halo cells stained as described above were imaged as follows: Eight line accumulations were performed, each of the following sequence: both Condensin II (CAP-H2–mEGFP, Alexa Fluor 594; 594-nm excitation laser, 30% [3.6 µW]) and Condensin I (CAP-H–Halo, Abberior STAR RED; 640-nm excitation laser, 40% [95.6 µW]) channels were accumulated five times (resulting in a total of 40 accumulations) and imaged superresolved (775-nm depletion laser, 40% [166.3 mW]) in line-sequential mode using 605–625-nm and 650–720-nm band pass filters, respectively, for detection on separate APDs. The pinhole was set to 1.2 airy units, and the pixel dwell time was set to 2 µs. The pixel size in xy was 20 nm. Z stacks of 13 slices and a z interval of 140 nm were acquired.

Similar to the single-color STED data, 0.1-µm TetraSpeck microspheres mounted on glass slides were imaged to align STED and excitation beams and to determine a potential offset between the superresolved immunostained Condensin I and Condensin II channels. If necessary, an offset was corrected before performing the colocalization analyses shown in Fig. 3 C.

### Analysis of STED data

#### Single-color STED image analysis: Condensin width and intensity distributions

ROIs containing straight or marginally bent chromatids mostly separable from neighboring chromatids were cropped for further analysis. A potential drift between consecutive z slices of the STED channel was determined by applying the MATLAB (R2017a; MathWorks) imregtform function using rigid transformation and multimodal optimization. The transformation function representing the drift was applied to correct both the STED channel of the immunostained Condensin-mEGFP and the diffraction-limited DNA channel. A potential offset between the Condensin and DNA channels was determined based on z stacks of 0.1-µm TetraSpeck microspheres using the channel alignment tool in ZEN 2012 Black software and applied to the DNA channel to align Condensin and DNA channels. An in house–developed MATLAB script was used to manually segment the 3D Condensin volume from the STED channel by drawing the outline of the Condensin region on individual z slices. The corresponding chromatid volume was segmented similarly from the DNA channel as shown in Fig. S3 A. Condensin and DNA z stacks as well as their binary masks were interpolated along the z dimension to achieve an isotropic voxel size in xy and z. The segmented binary mask of DNA was extended by including any voxels that were not segmented as DNA but as Condensin. The interpolated Condensin z stack was preprocessed using a 3D Gaussian filter (σ = 0.5).

All Condensin-positive voxels within the interpolated binary mask were represented by their three orthogonal eigenvectors and associated eigenvalues, where the largest eigenvector was used to cut the segmented Condensin volume at 100-nm spacing to generate a set of parallel cross sections orthogonal to the largest eigenvector. The centroids of each Condensin cross section were determined to adapt the direction of slicing based on the local vector generated from two neighboring centroids. Using these local vectors, both Condensin and DNA volumes were cut again at 20-nm spacing to generate cross sections compatible with the local curvature of the Condensin volume as shown in Fig. S3 A. The centroids of the final cross sections defined the Condensin axis (see Fig. S3 D, yellow line). The length of the Condensin axis per chromatid region was determined and used later for determining the number of Condensins per µm axis length. A small part of the Condensin volume from both ends of the chromatid was excluded from the analysis (see Fig. S3 A, gray).

To calculate the width of Condensin cross sections within a sliding window of 2-µm length, the cross sections were added up together, and a 1D profile was generated by taking the sum projection of the intensities along z as shown in Fig. S3 B. The width of Condensin was calculated by the FWHM of the projected intensity profile shown in Fig. S3 B. The sliding window was shifted by 200 nm to calculate the width of Condensin in a similar way. The mean of widths computed from all sliding windows per chromatid was taken as the Condensin width of this chromatid region. The chromatid width was calculated similarly. The Condensin width means of all chromatids per Condensin subunit and mitotic phase were combined to yield the means plotted in Fig. 3 A. The chromatid width means of all chromatids for all Condensin subunits per mitotic phase were combined to yield means for prometaphase and anaphase. Because the chromatid width in anaphase was not significantly different from the chromatid width in prometaphase, all chromatid width means independent of the Condensin subunit and mitotic phase were pooled to yield the mean plotted in Fig. 3 A.

To visualize the total intensity profile of Condensin subunits, all slices belonging to one chromatid region were added up to generate a 2D image (Fig. S3 C, leftmost panel) followed by sum projection. Profiles from all regions belonging to a particular Condensin subunit and mitotic phase were aligned to their maximum and merged to generate a combined profile. The profile was made symmetric by reflecting its left side to the right, taking the mean and reflecting it back to the left (see Fig. 3 B, bottom panels with total intensity profiles).

To generate the radial intensity profiles of Condensin subunits per mitotic phase, all cross sections belonging to one chromatid region were added up. Images from different regions belonging to a particular subunit and mitotic phase were combined by aligning them to a reference point at which the total intensity within a window size 0.4× and 0.6× the mean chromatid width in x and z, respectively, reaches its maximum (Fig. S3 C, second panel from left). An intensity profile through the reference point along the x axis (Fig. S3 C, second panel from left) was taken (Fig. S3 C, middle). The left side of the profile was reflected to the right to compute the mean representing the radial profile (Fig. S3 C, rightmost panel). For intuitive visualization, this profile was reconstructed into a 2D cross section (Fig. S3 C, fourth panel from left) in which the intensity from the center to the periphery at any angle corresponds to the 1D profile. The mean chromatid width was used to exclude Condensin intensities outside the chromatid (Fig. S3 C, white region in rightmost panel).

#### Double-color STED image analysis: colocalization analysis of Condensins I and II

Cropping of ROIs containing chromatid arms, drift correction between consecutive z slices, and correction of shift between the Condensin I and II channels were performed similarly to the single-color STED data. An initial manual segmentation of the combined Condensin volume was performed only in the Condensin I channel, and the same binary mask was applied to segment the Condensin II volume (see Fig. 3 C, first two panels). Colocalization analysis was performed by only considering the detected high-density voxels of Condensins I and II by further segmenting the initial combined Condensin volume. A global threshold for each Condensin was automatically detected by applying the Otsu method (Otsu, 1979). High-density voxels of Condensin signals were segmented by adapting the MATLAB Bradley local thresholding function (MATLAB Central, File Exchange; Bradley and Roth, 2005), whereby the global threshold was combined with a local threshold for a window of size 9 × 9 pixels to reduce the amount of over/undersegmentation. The percentage of nonoverlapping voxels was calculated by the number of nonoverlapping voxels divided by the geometric mean of the number of Condensin I– and Condensin II–positive voxels multiplied by 100. Condensin I– and Condensin II–positive voxels were combined to determine the intensity correlation between Condensin I and II using normalized cross correlation.

#### Single-color STED image analysis: automatic spot detection, clustering, counting, centroid determination, and coordinate-based distance measurements

Intensity peaks in each slice of a Condensin STED z stack were localized with FIJI (ImageJ; Schindelin et al., 2012) using the ThunderSTORM v1.3 plugin (https://github.com/zitmen/thunderstorm; Ovesný et al., 2014) with the following camera settings: readoutnoise = 0.0, offset = 0.0, quantumefficiency = 1.0, isemgain = false, photons2adu = 1.0, and pixelsize = 20.0 nm. Background noise was removed with a B-spline wavelet filter with an order of 2 and a scale of 4 followed by an approximation of peak position using a local maximum threshold of 3*SD (Wave.F1). The subpixel positions of the intensity peaks were found with the following settings: estimator = PSF: Integrated Gaussian, sigma = 2.2, fitradius = 2, method = maximum likelihood, full_image_fitting = false, and mfaenabled = false.

For each region, the detected x, y, and z positions within the manually segmented Condensin region (spots) were clustered using density-based spatial clustering of applications with noise (DBSCAN; v1.1-1 algorithm [Ester et al., 1996] in RStudio v3.4.2) with an eps value of 45 and minPts set to two. The z values were scaled by a factor of 1:8 to account for the ≥8 times poorer resolution in z (diffraction limited) in comparison with xy (STED superresolved) because of the anisotropy of 2D STED and to make peaks adjacent in z more likely to be placed in the same cluster. For the least abundant Condensin II subunit CAP-H2, most clusters were well separated from each other and assumed to represent one Condensin subunit and thus one Condensin complex. However, to account for the possibility of several Condensin subunits being present in the same high-intensity cluster, the cluster number per micrometer was corrected by not allowing clusters to be >3,000 arbitrary intensity units, thus using 3,000 arbitrary intensity units or multiples thereof as threshold for counting two or more Condensin subunits per cluster. The number of clusters per micrometer of Condensin axis length was calculated from the Condensin axis length per chromatid region (determined in the first subsection of the Analysis of STED data section) and the intensity-corrected number of clusters. The mean number of clusters representing the number of CAP-H2 subunits for all respective chromatid regions in prometaphase and anaphase was determined by averaging among all CAP-H2 chromatid regions per mitotic phase.

The performance of the outlined procedure of automatic spots counting, clustering, and intensity correction to account for multiple proteins per cluster was confirmed by manual spot counting of three chromatid regions of CAP-H2 in early anaphase using Imaris (v9.0.1; Bitplane), demonstrating a maximum deviation of the automated spots counting of 18% from manual counting.

Cluster centroids were determined and used for the following distance measurements: Pairwise distances between all cluster centroids were calculated using the MATLAB Inter-Point Distance Matrix function (MATLAB Central, File Exchange; https://nl.mathworks.com/matlabcentral/fileexchange/18937-ipdm-inter-point-distance-matrix), and the minimum distance of neighboring clusters was taken as the 2D nearest neighbor distance (NN; Fig. S3, D and E). To calculate the distance between cluster centroids and the Condensin axis, a distance-transformed image of the Condensin axis was generated in which the intensity of each pixel represents its distance from the axis. Thus, the coordinates of the cluster centroids were used to determine their distances from the Condensin axis (CA; Fig. S3, D and F). Cluster centroids were projected orthogonally on the Condensin axis to obtain a 1D representation of the distribution. The distance between neighboring 1D-projected cluster centroid pairs on the Condensin axis was calculated and used as readout for the axial spacing of Condensins (AS; Fig. S3, D and G).

### Airyscan imaging of live SMC4-mEGFP cells and determination of whole-cell chromatid length

2.5 × 10^4^ HK SMC4-mEGFP cells that had been passaged the day before were seeded into individual chambers of a Nunc eight-well LabTek II chambered coverglass (1.5; 155409; Thermo Fisher Scientific) and incubated ON at 37°C and 5% CO_2_ in a cell culture incubator. 2 h before imaging, the medium was changed to imaging medium (see the first subsection of Cell line validation) containing 50 nM SiR-DNA to stain the DNA, and cells were kept at 37°C until imaging.

Experiments were performed on an LSM880 laser-scanning microscope with an inverted Axio Observer operated by the ZEN 2.3 Black software with the AiryscanFast imaging option. The microscope was equipped with a temperature-controlled incubation chamber (constructed in house). Although live cells were imaged, the temperature was set to RT to slow down cell division and reduce cellular movement during the acquisition of individual z stacks. Images were acquired using a C-Apochromat 40× 1.20 W Korr FCS M27 water-immersion objective. Cells in late prometaphase, metaphase, or early anaphase were manually selected based on their DNA and Condensin signals. SMC4-mEGFP was excited with 488 nm (argon laser at 3.5%), and SiR-DNA was excited with 633 nm (HeNe laser at 2.8%). Detection was performed on the Airy disk consisting of 32 GaAsP photomultiplier tube detectors by using combined 495–550-nm band pass and 570-nm low pass filters. The AiryscanFast imaging mode was used. The xy pixel size was 50 nm. A z stack with a z interval of 200 nm of a manually defined size was acquired to cover the complete DNA volume of a cell. Airyscan z stacks were Airyscan processed in 3D using the ZEN 2.3 Black software and automatic settings.

The total chromosome length of a mitotic cell was computed by using the length of a small presegmented Condensin volume, the ratio of the total intensity of Condensin signal within the entire chromosomal volume, and the total intensity of the segmented Condensin volume. To this end, the SMC4-mEGFP channel was used to quantify the Condensin intensity, and the SiR-DNA channel was used to segment the chromosomal volume. The shift between the two channels was determined for individual cells by cross correlation within a 350-nm × 350-nm × 600-nm neighborhood and applying the mean offset among all cells to the DNA channel of all cells. A custom-written MATLAB script was used to manually segment a small Condensin volume largely distinguishable from other regions (Fig. 4 A). Individual z slices were preprocessed using a 2D Gaussian filter (σ = 2.5 for DNA, and σ = 2.0 for Condensin). DNA and Condensin signals were segmented by combining local and global thresholds as described previously (Hériché et al., 2014). The segmented slice containing the maximum number of Condensin-positive voxels was dilated using the diamond shape structuring element of 9-pixel radius and eroded using diamond shape structuring element of 7-pixel radius to extract the rim for quantifying Condensin background intensity. A histogram of the rim intensities was created, and the mean background intensity was calculated from the histogram without considering the lowest 10% and the highest 50% voxels.

The segmented DNA volume was applied to the Condensin signal and the manually segmented Condensin volume to exclude Condensin voxels outside the chromosomal volume. The manually segmented Condensin region was interpolated to yield isotropic voxel sizes in xy and z. Boundary voxels were detected by morphological operation, and their pairwise distances were calculated using the MATLAB Inter-Point Distance Matrix function. The pair of voxels with the largest distance was selected to determine the vector for slicing orthogonal cross sections of the manually segmented Condensin volume at 50-nm spacing. The centroids of each cross section were determined to define a central axis of the segmented Condensin volume. The length of the central axis was computed and considered as the length of the segmented Condensin volume. The total chromosome length of each cell was calculated from the total intensity of the Condensin signal within the entire DNA volume divided by the total intensity of the manually segmented Condensin volume multiplied by its Condensin axis length. Three to five Condensin regions per cell were manually segmented and used for determining the total chromatid length per cell. For each cell, the mean based on the calculations from different segmented Condensin regions was determined. For each mitotic phase, the mean of total cellular chromatid length was determined by averaging among three cells.

### Statistical methods

The relative precision of FCS-calibrated confocal imaging was determined by comparing the quantification of proteins on chromatin for cells in metaphase. The intra-assay variability given by the coefficient of variation (CV; CV = SD/mean) was ∼11%. This error accounted for the biological variability, precision of the imaging, and image analysis pipeline. The interassay variability, computed by calculating the mean and SD between all experiments, yielded a CV of 19.6%. This CV also accounted for the variability generated by the FCS calibration method.

For the FCS-calibrated imaging data and the FRAP data in Figs. 1 and S2, means, medians, SDs, and interquartile ranges were computed using R (https://www.r-project.org/) and ggplot. Robust linear regression (rlm function in the R package MASS) was used to compute the FCS calibration curve for each experiment (e.g., Fig. S1 G). To fit the exponential model to the FRAP data (Fig. S2, J–L), the Levenberg-Marquardt algorithm was used as implemented in the function nlsLM from the R package minpack.lm. Parameter distributions were computed by bootstrap (Efron and Tibshirani, 1994). To this end, a random sample containing the same number of cells as in the original dataset was created by random sampling with replacement. For each sampled set, the exponential model was fitted to the data. This operation was repeated *n* times (in this case, *n* = 300), yielding parameter distributions. From the parameter distributions, medians and interquartile ranges were computed. To compare the amount of CAP-H2 on metaphase chromosomes, the two-sided Kolmogorov-Smirnov test was used as implemented in the R function ks.test.

For the STED data in Fig. 3, means and SDs were calculated using MATLAB (R2017a; MathWorks). Significance tests of Condensin widths (Fig. 3 A) were based on Student’s two-tailed *t* tests and performed using Excel (2007; Microsoft). The following thresholds for p-values were used to indicate significance: NS, P ≥ 0.05; *, P < 0.05; **, P < 0.01; ***, P < 0.001.

Medians for nearest neighbor distance (NN), distance of cluster centroids from the central Condensin axis (CA), and axial spacing (AS) of CAP-H2 based on the coordinates of detected and clustered spots in the CAP-H2 STED data (Fig. S3, E–G) were calculated using MATLAB. Means and SD for whole-cell chromatid lengths (Fig. 4 B) were calculated using Excel.

### Online supplemental material

Fig. S1 shows the validation of genome-edited cell lines expressing homozygous mEGFP-tagged Condensin subunits as well as FCS-calibrated imaging methods. Fig. S2 shows the absolute quantification of Condensin-mEGFP subunits over mitosis by FCS-calibrated imaging and the results from FRAP experiments of Condensin-mEGFP subunits on the metaphase plate. Fig. S3 illustrates the methods to determine the width (FWHM) and intensity profiles of Condensin subunits from z stacks of chromatid regions acquired in 2D STED mode, the methods for automatic spot detection and clustering of CAP-H2 STED data, and distance measurements based on cluster centroids. Table S1 lists gRNA and ZFN sequences. Table S2 lists donor plasmids used for genome editing. Table S3 lists primers for junction PCR. Table S4 lists ABs used for WB. Table S5 lists probes used for SB. The source code for the image and data analysis methods can be found online. 

## Supplementary Material

Supplemental Materials (PDF)

Tables S1-S5 (ZIP)

Source Code (ZIP)
