# Supplementary material for: A quantitative map of human Condensins provides new insights into mitotic chromosome architecture
Source: J Cell Biol. 2018 Jul 2;217(7):2309–28. doi: 10.1083/jcb.201801048 (PMC6028534; doi:10.1083/jcb.201801048)
Supplement: Supplemental Materials (PDF) [file JCB_201801048_sm.pdf]

Supplemental material

Walther et al., https://doi.org/10.1083/jcb.201801048

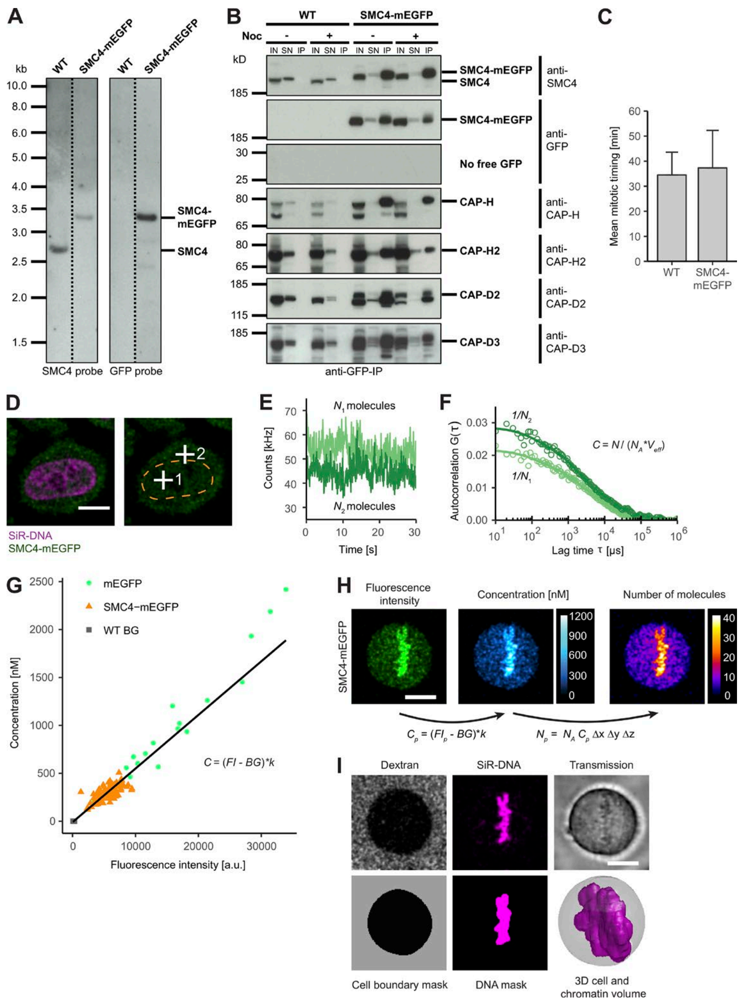

Figure S1. **Validation of genome-edited homozygous mEGFP knock-in cell lines and methods for FCS-calibrated confocal imaging and determination of protein numbers on mitotic chromosomes. (A–C)** Genome-edited HK cells homozygously mEGFP-tagged for Condensin subunits were validated for homozygosity as well as absence of both genomic rearrangements and extra integrations of the mEGFP donor by SB (A); anti-GFP IP of asynchronous (Noc$^-$) and mitotically arrested cells (Noc$^+$) as well as WB against the other Condensin subunits to ensure the incorporation of tagged subunits into functional pentameric Condensin complexes, homozygous expression of the mEGFP-tagged protein, and absence of the expression of free GFP (B); and nonperturbance of mitotic timing (prophase to anaphase onset; means + SD) by widefield time-lapse microscopy and automatic classification into mitotic phases (C). In A, the expected sizes for mEGFP-tagged and non-tagged SMC4 by the GFP and endogenous probes used for SB are 3.5 kb and 2.8 kb, respectively. In C, one representative experiment out of three independent experiments is shown (WT, *n* = 87; SMC4-mEGFP, *n* = 71). Validation data in A–C are exemplarily shown for the homozygous HK SMC4-mEGFP cell line. All other homozygous Condensin-mEGFP cell lines used in this study were validated similarly. **(D–I)** Methods for FCS-calibrated confocal imaging and determination of protein numbers on mitotic chromosomes (details for Fig. 1, B–D). **(D)** FCS measurements were acquired in the nucleus (point 1) and cytoplasm (point 2) of cells expressing the POI tagged with mEGFP (e.g., SMC4-mEGFP), mEGFP alone, and WT cells that do not express mEGFP. At each FCS position, the fluorescence intensity (*FI*) was measured in an ROI of pixel size 9 × 9. HK interphase cells were expressing SMC4-mEGFP and stained with SiR-DNA. The nuclear border is indicated with an orange dashed line. **(E)** Photon counts at the positions indicated in D recorded over time. **(F)** Autocorrelation curves were computed (dots) and fitted to a two-component diffusion model (solid lines) to extract the number of molecules N_1 and N_2 in the focal volume. The concentration *C* was calculated by dividing the number of molecules with the effective focal volume V_{eff} and the Avogadro constant N_A. The effective focal volume has been determined from a measurement of the diffusion time of an Alexa Fluor 488 dye solution in the same experiment. D–F depict representative data from 414 FCS measurements from 140 SMC4-mEGFP cells (two to six FCS points per cell) and five experiments. On average, 426 FCS measurements from 141 cells were acquired per genome-edited cell line in five independent experiments. **(G)** The FCS-calibration curve obtained from measurements of cells expressing the POI-mEGFP (SMC4-mEGFP, *n* = 118 measurements from 36 cells), mEGFP alone (*n* = 19 measurements from 12 cells), or WT cells (*n* = 10 measurements from five cells) for background (BG) correction. Concentrations are plotted against the fluorescence intensities and fitted to a linear function (straight line indicated). Representative data from one experiment are shown. **(H)** The pixel fluorescence intensity (FI_p; left) is converted to concentrations (middle) by applying the linear relationship from G and protein numbers (right panel) by multiplying with the voxel volume $\Delta x\, \Delta y\, \Delta z$ and the Avogadro constant N_A. An SMC4-mEGFP metaphase cell is shown. **(I)** Exemplary segmentation of cell boundary (left) and DNA (middle) based on extracellular fluorescently labeled Dextran or SiR-DNA staining, respectively, of the SMC4-mEGFP cell shown in H. A transmission image as well as the 3D-reconstructed cell (gray) and chromatin (magenta) volumes are shown on the right. Bars, 10 µm.

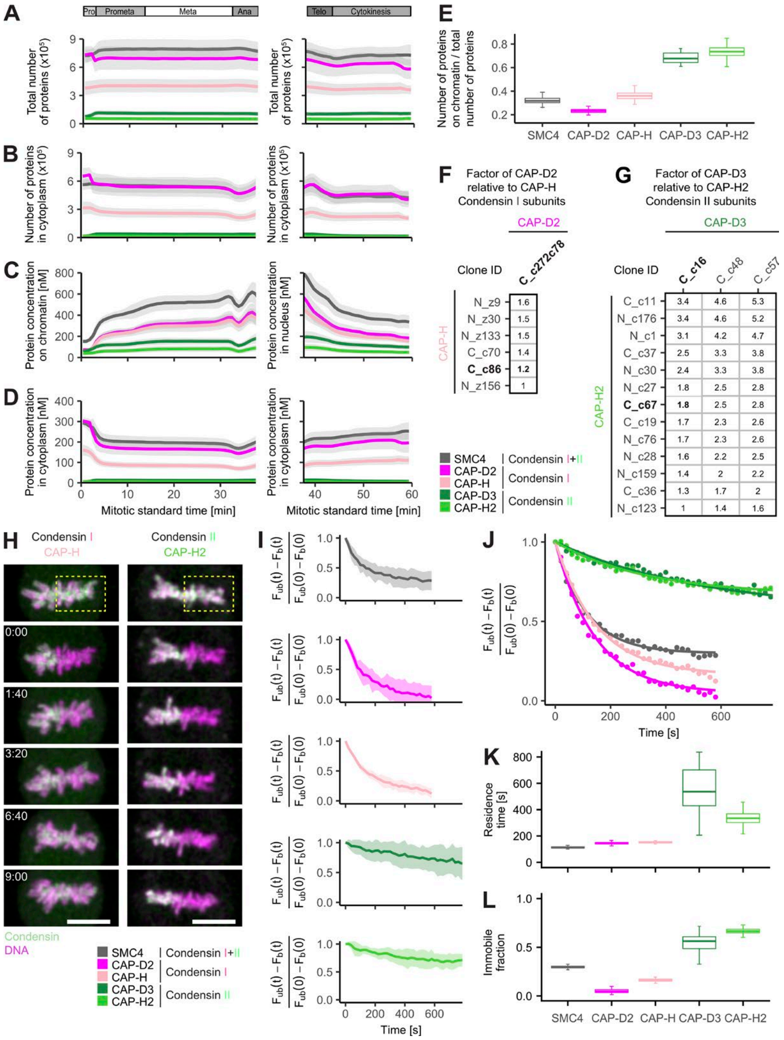

Figure S2. **Quantitative live-cell data of Condensin subunits in mitotic cells and FRAP of Condensin subunits on the metaphase plate. (A–G)** Quantitative live-cell data of HK cells with homozygously mEGFP-tagged Condensin subunits (details for FCS-calibrated imaging in Fig. 1): SMC4 (Condensin I/II, gray), CAP-D2 (dark magenta), and CAP-H (light magenta, Condensin I) as well as CAP-D3 (dark green) and CAP-H2 (light green, Condensin II). **(A)** Total protein numbers as function of the mitotic standard time. **(B)** Number of proteins in the cytoplasm. This was computed by summing up all proteins in the cytoplasmic region. After NEBD to the end of anaphase, Eq. 3 (see Materials and methods) was used. **(C)** Concentration of proteins bound to chromosomes (left) or in the nucleus (right). Before NEBD, Eq. 5 was used, and after NEBD up to the end of anaphase, Eq. 1 was used. **(D)** Concentration of proteins in the cytoplasm. Means (colored lines) and SD (light gray areas) are shown (~20 cells per subunit [range, 10–36] with three to seven independent experiments; 36, 22, 10, 17, and 17 cells from seven, three, five, three, and four experiments for CAP-H, CAP-H2, CAP-D2, CAP-D3, and SMC4, respectively). **(E)** Fraction of proteins bound to chromatin measured in metaphase (Fig. 1, C and D). The median, upper, and lower quartiles as well as 1.5× the interquartile range are shown (~20 cells per subunit [range, 10–36] with three to seven independent experiments; 36, 22, 10, 17, and 17 cells from seven, three, five, three, and four experiments for CAP-H, CAP-H2, CAP-D2, CAP-D3, and SMC4, respectively). **(F)** Fold difference in the Condensin I subunits bound to chromatin between CAP-H and CAP-D2 for different homozygous clones. For CAP-H, both C- and N-terminally mEGFP-tagged clones are shown. The clones used in this study are indicated in bold. The number of cells is 22, 7, 6, 5, 19, and 2 for CAP-H N_z9, N_z30, N_z133, C_c70, C_c86, and N_z156, respectively, and 26 for CAP-D2 C_c272c78. **(G)** Fold difference in the Condensin II subunits bound to chromatin between CAP-H2 and CAP-D3 for different homozygous clones. For CAP-H2, both C- and N-terminally mEGFP-tagged clones are shown. The clones used in this study are indicated in bold. The number of cells is 7, 9, 24, 7, 10, 10, 14, 7, 6, 7, 8, 6, and 7 for CAP-H2 C_c11, N_c176, N_c1, C_c37, N_c30, N_c27, C_c67, C_c19, N_c76, N_c28, N_c159, C_c36, and N_c123, respectively, and 18, 8, and 6 for CAP-D3 C_c16, C_c48, and C_c57, respectively. **(H–L)** FRAP experiments of Condensin subunits on the metaphase plate. **(H)** FRAP of mEGFP-tagged Condensin subunits (green) was performed by bleaching half of the metaphase plate (chromosomes stained by SiR-DNA) at time 0 as indicated in the prebleach image ROI (yellow boxes) and acquiring a time course (Δt = 20 s). Maximum projected images of z planes 3–7 are shown. A Gaussian blur (σ = 1) was applied to the images for presentation purposes. Bars, 10 µm. The images are representative for n = 9 (CAP-H) and n = 15 (CAP-H2) cells from two and three independent experiments, respectively. **(I)** Normalized fluorescence intensity difference in the unbleached (F_{ub}) and bleached (F_b) chromatin region. Shown are means ± SD of n = 16 (SMC4; gray), n = 14 (CAP-D2; dark magenta), n = 9 (CAP-H; light magenta), n = 15 (CAP-D3; dark green), and n = 15 (CAP-H2, light green) cells. Two to four independent experiments per Condensin subunit were acquired. **(J)** The mean of fluorescence recovery traces (points) and fits to Eq. 7 (solid lines) are shown for each subunit. **(K)** The residence time τ was defined by Eq. 8 and plotted for the different Condensin cell lines (median, upper, and lower quartile as well as 1.5× the interquartile range). **(L)** The immobile fraction was defined by Eq. 7 and plotted for the different cell lines (median, upper, and lower quartiles as well as 1.5× the interquartile range). The data plotted in J–L are based on two to four independent experiments with n = 16 (SMC4; gray), n = 14 (CAP-D2; dark magenta), n = 9 (CAP-H; light magenta), n = 15 (CAP-D3; dark green), and n = 15 (CAP-H2, light green) cells. The statistics were computed by bootstrapping the data (for details, see Materials and methods).

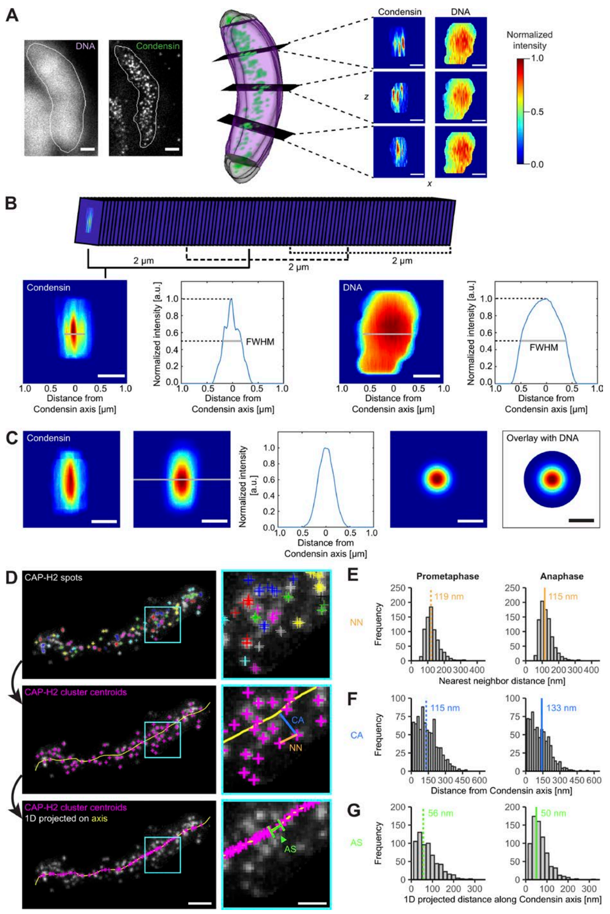

Figure S3. **Analysis of Condensin STED data to determine the subchromosomal distribution of Condensin subunits and automatic spot detection for distance measurements of the Condensin II subunit CAP-H2. (A–C)** Subchromosomal distribution of Condensin subunits based on 3D STED data of Condensin subunits in HK cells. **(A)** Z stacks of immunostained mEGFP-tagged Condensin subunits were acquired by superresolution microscopy in 2D STED mode. Chromosomes stained by Hoechst were acquired by diffraction-limited microscopy. Left: Condensin and chromatid were segmented manually, slice by slice. A representative z plane of a CAP-D3 chromatid in anaphase is shown. Right: 3D visualization of Condensin (green) in the segmented DNA volume (magenta). Condensin and DNA channels were interpolated along z to achieve an isotropic voxel size in x, y, and z. The central Condensin axis was determined, and cross sections along the chromatid diameter and perpendicular to the Condensin axis compatible with the local curvature of the Condensin volume were selected. Zooms of cross sections and the corresponding normalized intensities of the Condensin and DNA channels are provided. **(B)** To calculate the width of Condensin, cross sections within a sliding window of 2-µm length were added up together, and a 1D profile was generated by taking the sum projection of the intensities along z. The width of Condensin was calculated by the FWHM of the projected intensity profile. The sliding window was shifted by 200 nm repeatedly, the Condensin width was calculated in a similar way for all 2-µm sliding windows, and the Condensin width of the chromatid region was taken as the mean among all sliding windows. The Condensin width of one particular subunit and mitotic phase was calculated by taking the mean from all chromatid regions belonging to this particular subunit and mitotic phase. The chromatid width was calculated similarly. **(C)** To visualize the total Condensin intensity profile per chromatid region, all cross sections belonging to one Condensin region were added up to generate a combined 2D image (leftmost panel). Combined images calculated from different regions belonging to a particular subunit and mitotic phase were combined by aligning them to a reference point. An intensity profile through the reference point along the x axis (second panel from left) was plotted (middle). The left side of the profile was reflected to the right to compute the mean intensity profile representing a 1D radial profile. For intuitive visualization, this profile was reconstructed into a 2D cross section in which the intensity from the center to the periphery at any angle corresponded to the 1D profile (fourth panel from left). The mean chromosome width was used to exclude the profile outside the chromosome marked in white (rightmost panel; see radial intensity profiles in the top panels of Fig. 3 B). In A and B, the same CAP-D3 chromatid in anaphase representative for $n = 20$ chromatids is shown. In C, mean data from $n = 20$ CAP-D3 chromatids in anaphase are shown. Bars, 500 nm. **(D–G)** Automatic spot detection and clustering for distance measurements of the Condensin II subunit CAP-H2. **(D)** Spots in z stacks of CAP-H2 2D STED data were detected automatically and clustered (top; different colors indicate different clusters). The cluster centroids (magenta) were determined, and their nearest neighbor distances (NN; orange) as well as distances from the yellow central Condensin axis (CA; blue) were calculated in 2D (middle). Cluster centroids were projected onto the central Condensin axis, and their 1D distances representing their axial spacing (AS; green) were calculated (bottom). A representative prometaphase chromatid of CAP-H2 is shown, with overview images on the left and zooms (turquoise boxes) on the right. Bars: (overviews) 500 nm; (zooms) 200 nm. **(E)** Histograms of the nearest neighbor distance of cluster centroids of CAP-H2 are plotted for prometaphase (left) and anaphase (right). The median is plotted as an orange line (dashed for prometaphase and continuous for anaphase), and the exact value is indicated. **(F)** Histograms of the distance of cluster centroids of CAP-H2 from the central Condensin axis are plotted for prometaphase (left) and anaphase (right). The median is plotted as a blue line (dashed for prometaphase and continuous for anaphase), and the exact value is indicated. **(G)** Histograms of axial spacing of cluster centroids of CAP-H2 projected in 1D onto the central Condensin axis are plotted for prometaphase (left) and anaphase (right). The median is plotted as a green line (dashed for prometaphase and continuous for anaphase), and the exact value is indicated. Prometaphase, $n = 12$; anaphase, $n = 16$.

Table S1 is a separate Excel file showing gRNA and ZFN binding sequences for genome editing.

Table S2 is a separate Excel file showing donor plasmids for genome editing.

Table S3 is a separate Excel file showing primers for junction PCR.

Table S4 is a separate Excel file showing antibodies for WB.

Table S5 is a separate Excel file showing probes for SB.

The source code for the image and data analysis methods can be found online. MitoSys contains and links to the source code for analyzing FCS-calibrated confocal time-lapse data through mitosis and generating time-resolved cellular protein concentration and number maps (Fig. 1, B–D; and Fig. S2, A–E). Segmentation_single_cell contains source code used to segment chromosomes in metaphase cells (Fig. S2, F and G). FRAP contains the source code for analyzing FRAP data (Fig. S2, I–L). Chromatid_structure_analysis contains the source code for the analysis of single-color (Fig. 3, A and B) and double-color (Fig. 3 C) Condensin STED data, the distribution analysis of Condensin spots (Fig. S3, E–G), and the determination of whole-cell chromatid length (Fig. 4, A and B). zStackSpotPicker contains the source code for detecting and clustering Condensin spots (Fig. S3 D). The latest version of the source code can also be found at https://git.embl.de/grp-ellenberg/condensin_map_walther_jcb_2018.
