# Supplementary material for: A quantitative map of human Condensins provides new insights into mitotic chromosome architecture
Source: J Cell Biol. 2018 Jul 2;217(7):2309–28. doi: 10.1083/jcb.201801048 (PMC6028534; doi:10.1083/jcb.201801048)
Supplement: Source Code (ZIP) [file JCB_201801048_DataS1.zip › WaltherEtAl_SourceCode/WaltherEtAl_zStackSpotPicker.zip!/zStackSpotPicker/ExampleData/Raw_Images_IJ_20180206_DBSCAN_20180306_plot_20180306/c0020_r0001_Cond2-H2_2dplot02.pdf]

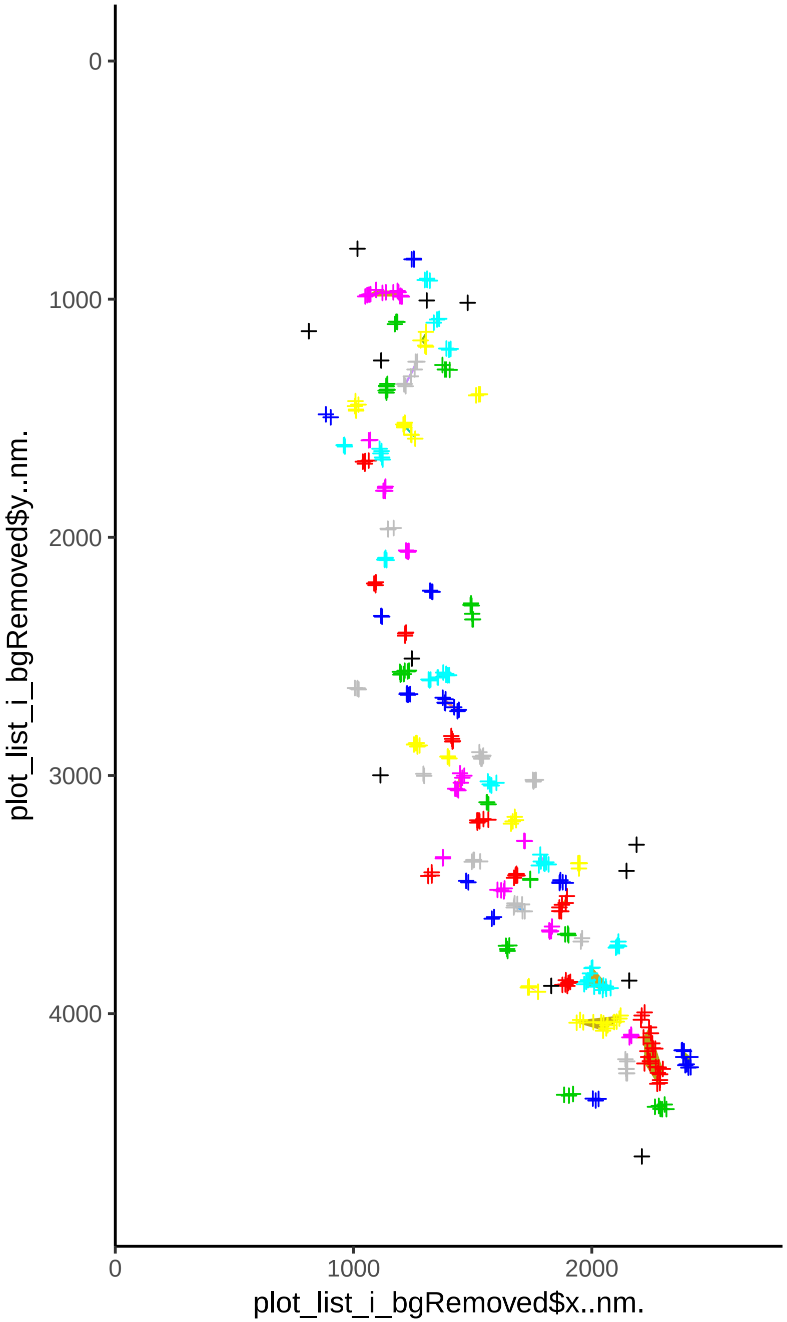

plot_list_i_bgRemoved$y..nm.
0
1000
2000
3000
4000
plot_list_i_bgRemoved$x..nm.
0
1000
2000
